# Mitochondrial diabetes mellitus: real world insights from the GENOMIT registry–a multinational, longitudinal cohort study

**DOI:** 10.1016/j.ebiom.2026.106458

**Published:** 2026-09-01

**Authors:** Luisa Semmler, Boriana Büchner, Cornelia Kornblum, Marcus Deschauer, Saskia Wortmann, Chiara La Morgia, Serenella Servidei, Peter Freisinger, Alexander Mensch, Markus Schuelke, Holger Prokisch, Michelangelo Mancuso, Costanza Lamperti, Thomas Klopstock, GENOMIT study group, Enrico Bertini, Almut T. Bischoff, Nikolas Boy, Claudio Bruno, Valerio Carelli, Giulia Cecchi, Kristl Claeys, Felix Distelmaier, Massimiliano Filosto, Caterina Garone, Maja Hempel, Daniela Karall, Chirstian Kmop, Rania Kotzaeridou, Piervito Lopriore, Tiziana Mongini, Vincenzo Montano, Olimpia Musumeci, Valeria Nicoletta, Guido Primiano, Elena Procopio, Rita Rinaldi, Lucia Ruggiero, René Santer, Heinrich Sasse, Jochen Schäfer, Christian Schlein, Ludger Schöls, David Strube, Annemarie Thaele, Maria Lucia Valentino, Jürgen-Christoph von Kleist, Leopold Zeng

**Affiliations:** a Department of Neurology, TUM-University Hospital, Munich, Germany; b Institute of Human Genetics, TUM-University Hospital, Munich, Germany; c Institute of Neurogenomics, Computational Health Centre, Helmholtz Munich, Neuherberg, Germany; d Friedrich-Baur-Institute at the Department of Neurology, LMU University Hospital, LMU Medizin, Ludwig-Maximilians-Universität München, Munich, Germany; e Department of Neuromuscular Diseases, Centre for Neurology, University Hospital Bonn, Bonn, Germany; f University Children’s Hospital, Paracelsus Medical University, Salzburg, Austria; g IRCCS Istituto delle Scienze Neurologiche di Bologna, Programma di Neurogenetica, Bologna, Italy; h Department of Biomedical and Neuromotor Sciences, University of Bologna, Bologna, Italy; i Dipartimento di Neuroscienze, Organi di Senso e Torace, Fondazione Policlinico Universitario Agostino Gemelli Irccs, Roma, Italy; j Klinikum am Steinenberg, Children’s Hospital Reutlingen, Reutlingen 72764, Germany; k Department of Neurology, University Medicine Halle, Halle (Saale), Germany; l BG University Hospital Bergmannsheil, Heimer Institute for Muscle Research, Bochum, Germany; m Department of Neuropediatrics, Charité-Universitätsmedizin Berlin, Corporate Member of Freie Universität Berlin, Humboldt-Universität Berlin, and Berlin Institute of Health, Berlin, Germany; n German Centre for Child and Adolescent Health (DZKJ), partner site Munich, Munich, Germany; o Department of Clinical and Experimental Medicine, Neurological Institute, University of Pisa, Pisa, Italy; p UO di Genetica Medica e Neurogenetica, Fondazione IRCCS Istituto Neurologico C. Besta, Milan, Italy; q Munich Cluster for Systems Neurology (SyNergy), Munich, Germany; r German Centre for Neurodegenerative Diseases (DZNE), Munich, Germany

**Keywords:** Mitochondrial disease, Diabetes mellitus, Mitochondrial diabetes, Metformin, m.3243A>G

## Abstract

**Background:**

Diabetes mellitus is a common but incompletely characterised manifestation of mitochondrial diseases (MD). Data on risk factors, clinical course, and treatment recommendations are lacking.

**Methods:**

In this multinational cohort study, we analysed longitudinal data of patients with a genetically confirmed MD from the GENOMIT registry included at German, Austrian, and Italian sites between 07/2009–01/2025. Our objectives were to (1) expand the genetic spectrum of mitochondrial diabetes mellitus (mDM), (2) identify risk factors, (3) delineate the clinical course, and (4) characterise real-world use of antidiabetic therapies.

**Findings:**

Of 2399 patients, 1225 (51%) were female, and 281 (12%; 172 female) had mDM. Diabetes occurred across 31 genotypes and exhibited marked genotype dependence, with the highest prevalence in m.3243A>G carriers (177/360 [49%]). Only the m.3243A>G variant was associated with a significantly increased risk of mDM (HR = 10.3; 95% CI 5.2–20.4, p < 0.0001), whereas single mtDNA deletions, multiple mtDNA deletions, and primary LHON variants, as well as sex, BMI, ethnicity, smoking, hypertension and dyslipidaemia did not show a significant association. Median diabetes onset in patients with the m.3243A>G variant was at 47.7 years (SD 45.2–52.0). Among patients with mDM, 140/281 (50%) used insulin, and 111/281 (40%) received non-insulin antidiabetic drugs, most commonly metformin, which was discontinued in 8/50 users. Literature review revealed neurological events temporally linked to metformin application in m.3243A>G carriers, though long-term use without adverse events was likewise reported.

**Interpretation:**

mDM is frequent in patients with MD, and the individual risk is strongly genotype dependent. While caution is warranted, our data do not justify universal avoidance of metformin; prospective, genotype-informed studies are needed to guide management.

**Funding:**

German Ministry of Research, Technology and Space; Italian Ministry of Health; European Union.

**Research in context**

**Evidence before this study**

We performed bibliographic searches between 1 January 2025 and 31 December 2025 using Ovid MEDLINE and PubMed. We used the search terms “mitochondrial diabetes” OR “mitochondrial disease” AND metformin OR “glucagon-like peptide 1” OR GLP-1 OR “dipeptidyl peptidase 4” OR DPP-4 OR “sodium-glucose cotransporter 2” OR SGLT2 OR sulfonylurea OR insulin). In PubMed, we used the MeSH terms “Mitochondrial Diseases” AND “Hypoglycemic Agents.” Mitochondrial diabetes mellitus (mDM) is a recognised but incompletely characterised manifestation of mitochondrial diseases (MD). Available evidence is largely limited to single case reports and small cohorts, leaving uncertainty about risk factors, genotype-specific prevalence, natural history, and complication burden. Treatment guidance is sparse; in particular, metformin is often discouraged because of its inhibitory effects on respiratory-chain complex I, complicating therapeutic decision-making in this population.

**Added value of this study**

In this multicentre, multinational cohort study of 2399 individuals with a genetically confirmed MD, we provide real-world data on prevalence, risk factors, clinical course, and treatment approaches of mDM. We identify additional genotypes associated with diabetes and show marked genotype dependence of diabetes prevalence, reaching 49% in m.3243A>G carriers. We characterise disease severity and progression, provide evidence of microvascular end-organ damage, and describe treatment patterns with a high rate of insulin dependence (50% of patients). Over one third of patients (40%) received oral or subcutaneous (non-insulin) antidiabetic drugs; in our cohort, and supported by our review of the literature, metformin exposure was not associated with an increased risk of adverse events in most patients, including individuals treated for many years, arguing against a blanket contraindication despite necessary caution.

**Implications of all the available evidence**

Given the substantial burden of mDM, systematic screening and early diagnosis should be prioritised in people with MD, particularly with high-risk genotypes. Management should be individualised, balancing glycaemic control, comorbidities, and mitochondrial-specific risks. Our findings support the need for prospective clinical trials of glucose-lowering agents, including metformin, in this patient population.

## Introduction

Mitochondrial diseases (MD) are genetically heterogeneous disorders of impaired energy metabolism caused by pathogenic variants in nuclear DNA or mitochondrial DNA (mtDNA).^1^^,^^2^ With a prevalence of around 1:4300, MD are the most common inherited neurological disorder in adults, accounting for a significant proportion of global disease-related burden, morbidity, and mortality.^3^ Mitochondrial diabetes mellitus (mDM) is the most common yet incompletely characterised endocrine manifestation of MD.^4^ The American Diabetes Association classifies most diabetes mellitus (DM) into type 1 (T1DM), type II (T2DM), or gestational; mDM is categorised among “other specific types of DM”. This group includes drug- or chemical-induced DM as well as several inherited forms such as maturity-onset diabetes of the young.^5^ mDM occurs across multiple MD genotypes, most commonly in association with the mtDNA m.3243A>G variant,^4^^,^^6^ which can manifest in different ways, including the syndromes maternally inherited diabetes and deafness (MIDD) and mitochondrial encephalopathy, lactic acidosis and stroke-like episodes (MELAS).^7^ The clinical course of mDM is heterogeneous, and prior descriptions–largely focused on common MD genotypes–remain limited.^8^^,^^9^ Proposed mDM pathomechanisms include impaired insulin secretion, insulin resistance, or both, but remain unresolved.^8^^,^^10^, ^11^, ^12^ Treatment guidance is sparse; concerns about metformin, a respiratory chain complex I inhibitor, and the potential risk of lactic acidosis have complicated therapeutic decisions.^13^^,^^14^ Real-world data from large cohorts are lacking. Given the high morbidity and mortality associated with DM,^15^ a precise characterisation of mDM is needed to enable risk stratification and to inform prevention and therapy in this vulnerable population. We analysed longitudinal data (2009–2025) from 2399 individuals with a genetically confirmed MD enrolled in the international GENOMIT registry (www.GENOMIT.eu). Our objectives were to (1) define the genetic spectrum of mDM; (2) identify risk factors for mDM, including anthropometric measures, social and ethnic background, lifestyle factors, and mtDNA heteroplasmy; (3) delineate the clinical course, emphasising determinants of early onset and insulin dependence; and (4) describe real-world use of antidiabetic therapies in this cohort and, together with a comprehensive literature review, inform the development of treatment recommendations.

## Methods

### Study design and participants

We analysed longitudinal data collected prospectively from July 2009 to January 2025 within the GENOMIT international mitochondrial disease registry. The analytic cohort comprised 3441 individuals with MD enrolled at GENOMIT centres in Germany and Austria (n = 2078), and Italy (n = 1363).

### Ethics

All participants (or their legal guardians) provided written informed consent for registry participation. Ethics approval for data collection exists at each GENOMIT site. The present analysis was approved by the ethics committee of TUM University Hospital Munich (EC# 2024-17-S-CB and 2025-397-S-KK) and conducted in accordance with the Declaration of Helsinki (2024 revision).

### Procedures

#### Definition of mitochondrial diabetes mellitus

Overt mDM was defined a priori as a registry documentation of “diabetes mellitus” or related Human Phenotype Ontology terms recorded in any symptom section or a score ≥3 on the Newcastle Mitochondrial Disease Adult Scale (NMDAS)^16^ diabetes item (Table 1). Patients with registry entries of “hyperglycaemia” or “impaired glucose tolerance” or a score of 2 on the NMDAS diabetes item were defined as having impaired glucose tolerance (IGT); and those with entries of “gestational diabetes”, or “transient diabetes” or a score of 1 on the NMDAS diabetes item were attributed as having transient diabetes. Registry participants without any of the mentioned entries were classified as having no mDM.

**Table 1:** The Newcastle Mitochondrial Disease Adult Scale (NMDAS) section II question 7, and section III question 7.

Section II—system-specific involvement		
Item	Description	Score
7. Diabetes mellitus	None.	0
	Past history of gestational diabetes or transient glucose intolerance related to intercurrent illness.	1
	Impaired glucose tolerance (in absence of intercurrent illness).	2
	NIDDM (diet).	3
	NIDDM (tablets).	4
	DM requiring insulin (irrespective of treatment at onset).	5

Section III—Current Clinical Assessment (examination at time of assessment)		
Item	Description	Score
7. Neuropathy	None.	0
	Subtle sensory symptoms or areflexia.	1
	Sensory impairment only (e.g., glove & stocking sensory loss).	2
	Motor impairment (distal weakness) or sensory ataxia.	3
	Sensory ataxia or motor effects severely limit ambulation.	4
	Wheelchair bound primarily due to sensory ataxia or neurogenic weakness.	5

Each item is scored 0–5. The total score (Sections I–III) reflects phenotypic severity; higher scores indicate greater disease burden. The diabetes item (Section II, item 7) was excluded from total score calculations in this study. Abbreviations: NIDDM: non-insulin dependent diabetes mellitus.

#### Genotype

Genotypic classification was based on the molecular diagnosis recorded in GENOMIT, including inheritance pattern, gene, and variant. Individuals with no genotype provided or with entries not confirming MD (e.g., “normal genotype”, “variant of uncertain significance”) were excluded from all analyses. To increase statistical robustness and interpretability of the data, we formed five main genotype groups for the analytical part of the study: “m.3243A>G”, “multiple mtDNA deletions”, “single mtDNA deletion”, “primary Leber hereditary optic neuropathy (LHON) variant”, and “others”. The multiple mtDNA deletions group comprises patients with multiple mtDNA deletions, both with and without an identified nuclear defect including variants in “POLG”, “POLG2”, “TWNK”, “ANT1”, “DGUOK”, “TK2”, “SLC25A4”,”RRM2B”,”TYMP”, “OPA1”, “MFN2”, “DNA2”, “MGME1”, and “TOP3AW”. The “primary LHON variant” group encompasses patients carrying one of the variants “m.11778G>A”, “m.14484T>C”, “m.3460G>A”.

For patients with the m.3243A>G genotype, blood heteroplasmy levels were age-adjusted according to a published formula^17^:

$$corrected\;heteroplasmy=\frac{heteroplasmy\;\left(blood\right)}{0.977^{\left(age\;+\;12\right)}}$$

#### Phenotype, anthropometry, and social background

We extracted each participant’s phenotype, affected organ systems, body mass index (BMI), glycated haemoglobin (HbA1c), ethnicity, education level, smoking status, alcohol consumption, dietary regimens, and performance on clinical scales from the registry. When multiple visits were available for a participant, the most recent visit was used for analysis. We defined the term “MELAS spectrum” to encompass all patients with a phenotype recorded as “MELAS“ in the registry, including also those without the classical presentation of stroke-like episodes. Ethnicity was self-reported by the patients and certified by clinicians. HbA1c levels could only be reported for German and Austrian patients, as this information was not available for the Italian patients.

#### Clinical scales

For adults, we analysed the scores of the NMDAS and the Scale for the Assessment and Rating of Ataxia (SARA). The NMDAS is a validated semiquantitative rating scale developed specifically for patients with MD to assess symptoms across multiple domains, including neuromuscular, systemic, and current functioning.^16^ The total score is widely used as a measure of phenotypic severity, with higher scores indicating greater disease burden. The diabetes item was excluded from the total score calculation to avoid artificially inflating scores in the diabetes cohort. When multiple visits were available for a participant, the most recent visit was used for analysis. The respective paediatric scale, the Newcastle Paediatric Mitochondrial Disease Scale (NPMDS), was not analysed due to insufficient data availability.

#### Polyneuropathy, retinopathy, nephropathy, and cardiovascular risk factors

Binary phenotypes were defined a priori using registry entries. Polyneuropathy was considered present if any of the following were recorded: “Polyneuropathy”, “peripheral neuropathy”, “sensorimotor neuropathy”, “decreased sensory nerve conduction velocity”, or related terms; we also classified polyneuropathy as present for a NMDAS neuropathy item score ≥1 (Table 1). Retinopathy was considered present for any entry of “retinopathy” or “maculopathy”. Nephropathy was considered present for entries of “chronic kidney disease”, “renal impairment,” or “nephropathy.” To characterise cardiovascular risk, we queried the registry for terms indicating “dyslipidaemia” and “hypertension.”

#### Antidiabetic therapies and dietary interventions

The registry captures medications with start and (when applicable) discontinuation dates. Among participants with mDM, we extracted entries for dietary measures, oral antihyperglycaemic agents, insulin therapy, and subcutaneous non-insulin antidiabetic drugs. Medication exposure was summarised as recorded in the registry; where dates were available, initiation and discontinuation were used to describe patterns of use.

#### Literature review of antidiabetic therapy use in mDM

We searched Ovid MEDLINE and PubMed (from inception through July 2025). For Ovid MEDLINE, the query combined (“mitochondrial diabetes” OR “mitochondrial disease”) AND (metformin OR “glucagon-like peptide 1” OR GLP-1 OR “dipeptidyl peptidase 4” OR DPP-4 OR “sodium-glucose cotransporter 2” OR SGLT2 OR sulfonylurea OR insulin). In PubMed, we used the MeSH terms “Mitochondrial Diseases” AND “Hypoglycemic Agents.” We included case reports and original research articles that reported the use of antidiabetic therapies in adult patients with mDM or MD; reviews were excluded.

#### Statistical analysis

**Fig. 1:**
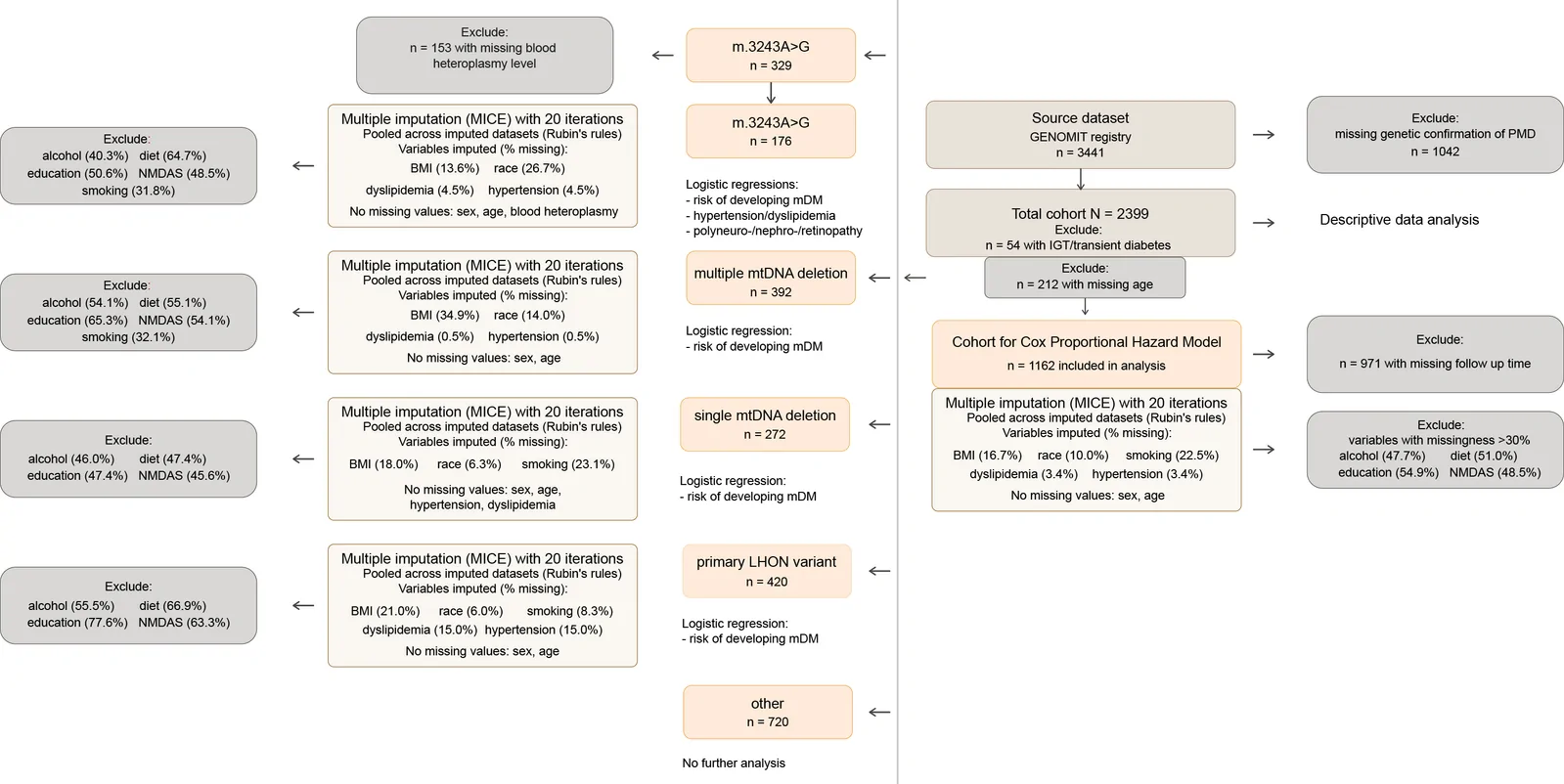
**Flowchart providing an overview of the analysed datasets, the missingness of variables, and the proportion of imputations.** Flowchart depicting the total study cohort (n = 3441), number of patients excluded (n = 1254) and stratification of different datasets with percentage of missing variables in each dataset. Variables with missingness ≥35% were excluded, cases with missing values for age, sex, and blood corrected heteroplasmy level were excluded. The R package mice was used for imputation for all other variables.

Analyses were performed in R (RStudio 2026.04.0 + 526; Posit, Boston, Massachusetts). Continuous variables are summarised as median (IQR) or mean (SD), as appropriate; categorical variables are presented as counts and percentages. Group comparisons used the Wilcoxon rank-sum test (Mann–Whitney) for continuous variables and Fisher exact test (with Monte Carlo simulation when required) for categorical variables. To control familywise error, p values were adjusted using the Bonferroni method; adjusted, 2-sided p values < 0.05 were considered statistically significant. To contextualise mDM prevalence, we calculated age-specific odds ratios (OR) for each genotype group relative to age-specific DM prevalence in the general German population using nationwide registry data.^18^^,^^19^ For the following inferential statistics, candidate covariates were specified a priori based on clinical relevance. Variables with >35% missingness were excluded from modelling. For the remaining missing data, multiple imputation by chained equations (*mice* package) was used resulting in 5 imputed datasets; estimates from imputed datasets were pooled using Rubin rules. An overview of the respective datasets, the missingness of their variables, and the imputed proportion is given in Fig. 1. We displayed differences in age of onset of mDM across genotype groups using a Kaplan–Meier curve, assessing significance with a log-rank test. We performed a Cox proportional hazards model to estimate hazard ratios (HR) for the development of mDM within the different genotype groups. We adjusted the models for potential confounders including sex, BMI, race, hypertension, dyslipidaemia, and smoking status. We report results as HR and 95% confidence intervals (CI). Model assumptions were assessed via Schoenfeld residuals (proportional hazards), likelihood ratio comparison of linear versus spline-modelled BMI (linearity), generalised variance inflation factors (multicollinearity), dfbeta residuals (influential observations), and the events-per-variable ratio (model stability). We fit multivariable logistic regression models to evaluate factors associated with (1) development of mDM in the genotype groups m.3243A>G, multiple mtDNA deletions, single mtDNA deletion, and primary LHON variants; (2) the influence of mDM on the occurrence of hypertension and dyslipidaemia; and (3) the association of mDM with the presence of polyneuropathy, retinopathy, and nephropathy. We assessed logistic regression assumptions, including linearity in the log-odds (Box-Tidwell), multicollinearity (GVIF), model calibration (Hosmer–Lemeshow), and events per variable (EPV).

### Role of funders

The funding sources had no role in the study design, data collection, data analyses, interpretation, writing or decision of submission. No payments have been received for writing the article. The authors were not precluded from accessing data in the study, and all accept responsibility to submit for publication.

## Results

### Population characteristics and prevalence of mDM

Of 3441 registry participants, 2399 with genetically confirmed MD were included in the analysis (1225 women and 255 children; 1274 from Germany and Austria, and 1125 from Italy); 1042 were excluded for lack of genetic confirmation. Among included participants, 281 (12%, 172 [61%] women) had overt mDM, 31 (1%, including 2 children) IGT, and 23 (1%) transient mDM. The remaining 2064 (86%) had no mDM and served as comparators. HbA1c was significantly higher in patients with mDM (Median [IQR], 7.0% [6.5–7.4]) compared to patients without mDM (Median [IQR], 5.4% [5.3–5.7]; Wilcoxon rank-sum test, p < 0.0001). Baseline characteristics of the total study population stratified by mDM status are shown in Table 2. A corresponding analysis restricted to the m.3243A>G genotype is provided in eTable 1. Assessing country differences, we found that the German mDM population was significantly taller, had a higher body weight, and an increased BMI compared to the Italian mDM population; further differences occurred in race distribution, education level, dietary regimens, and alcohol consumption, see eTable 2.

**Table 2:** Characteristics of study population.

Parameter	mDM	non mDM	total cohort	p value
Count [n]	281	2064	2399	
Age [yr]	57.7 (46.7–67.5)	42.5 (25.7–59.2)	45.2 (27.7–60.7)	<0.0001
Height [cm]	166 (159–170)	167 (156–175)	167 (158–175)	1.0
Weight [kg]	60 (50–74)	62.5 (47–77)	62 (48–76)	1.0
BMI [kg/m^2^]	22.1 (19.0–25.7)	22.0 (18.3–25.7)	21.97 (18.45–25.7)	1.0
HbA1c [%]	7.0 (6.5–7.4)	5.4 (5.3–5.7)	6.2 (5.5–7.0)	<0.0001
NMDAS [points]	19 (12–29.5)	13 (7–25)	14 (8–26)	<0.0001
SARA [points]	2.5 (0.5–7.5)	0.5 (0–5)	1 (0–6)	0.00051
Heteroplasmy in blood of m.3243A>G patients [%]	60.0 (44.9–98.0)	46.2 (22.7–86.9)	57.8 (29.8–86.6)	0.062
Age of PMD onset [years]	27.8 (18.1–45.8)	17.3 (4.3–33.8)	18.8 (6.3–36.2)	<0.0001
Time of follow-up [years]	3.3 (1.65–5.75)	3.26 (1.61–5.98)	3.31 (1.63–5.99)	1.0
Deaths [n (%)]	20 (7.1%)	132 (6.4%)	156 (6.5%)	1.0
Mortality rate [Deaths/1000 person years]	30.02	31.28	30.58	
Sex [n (%)]				0.00022
Female	172 (61.2%)	1021 (49.5%)	1193 (50.9%)	
Male	109 (38.8%)	1043 (50.5%)	1152 (49.1%)	
Race [n (%)]				0.003
White European descent	240 (96.0%)	1712 (93.2%)	1952 (93.5%)	
Other	1 (0.4%)	13 (0.7%)	14 (0.7%)	
Asian (other)	3 (1.2%)	86 (4.7%)	89 (4.3%)	
Multi-racial	1 (0.4%)	10 (0.5%)	11 (0.5%)	
Black (African)	1 (0.4%)	7 (0.4%)	8 (0.4%)	
Asian (Indian)	0 (0.0%)	4 (0.2%)	4 (0.2%)	
Asian (Chinese)	0 (0.0%)	3 (0.2%)	3 (0.1%)	
Hispanic	1 (0.4%)	2 (0.1%)	3 (0.1%)	
Native American	3 (1.2%)	0 (0.0%)	3 (0.1%)	
Education [n (%)]				0.0001
ISCED 0–early childhood education	0 (0.0%)	72 (10.0%)	72 (8.6%)	
ISCED 1–primary education	3 (2.6%)	68 (9.4%)	71 (8.5%)	
ICSED 2–lower secondary education	21 (17.9%)	116 (16.0%)	137 (16.3%)	
ISCED 3–upper secondary education	34 (29.1%)	164 (22.7%)	198 (23.6%)	
ISCED 4–post-secondary non-tertiary education	20 (17.1%)	102 (14.1%)	122 (14.5%)	
ISCED 5–short-cycle tertiary education	10 (8.5%)	61 (8.4%)	71 (8.5%)	
ISCED 6–Bachelor or equivalent	25 (21.4%)	77 (10.7%)	102 (12.1%)	
ISCED 7–Master or equivalent	1 (0.9%)	38 (5.3%)	39 (4.6%)	
ISCED 8–Doctoral or equivalent	2 (1.7%)	8 (1.1%)	10 (1.2%)	
ISCED 9–not elsewhere classified	1 (0.9%)	17 (2.4%)	18 (2.1%)	
Diet [n (%)]				0.0014
No diet (normal nutrition)	101 (72.7%)	652 (78.7%)	753 (77.9%)	
Other	10 (7.2%)	47 (5.7%)	57 (5.9%)	
Vegetarian	1 (0.7%)	11 (1.3%)	12 (1.2%)	
Ketogenic	0 (0.0%)	37 (4.5%)	37 (3.8%)	
Low protein	2 (1.4%)	3 (0.4%)	5 (0.5%)	
Low fat	1 (0.7%)	6 (0.7%)	7 (0.7%)	
Mediterranean	24 (17.3%)	72 (8.7%)	96 (9.9%)	
Smoking [n (%)]				0.029
No	165 (85.1%)	1096 (78.2%)	1261 (79.0%)	
Current smoker	11 (5.7%)	175 (12.5%)	186 (11.7%)	
Former smoker	15 (7.7%)	110 (7.8%)	125 (7.8%)	
Occasional smoker	3 (1.5%)	21 (1.5%)	24 (1.5%)	
Alcohol [n (%)]				0.27
No	113 (81.9%)	665 (74.5%)	778 (75.5%)	
Yes, daily	2 (1.4%)	29 (3.2%)	31 (3.0%)	
Yes, weekly	3 (2.2%)	41 (4.6%)	44 (4.3%)	
Yes, occasionally	20 (14.5%)	158 (17.7%)	178 (17.3%)	

Study population characteristics given for patients with MD with mDM and without mDM. We excluded the n = 53 patients with IGT and/or transient diabetes mellitus to prevent bias in the mDM, but also the control group. p values indicate significance of differences between groups. BMI: body mass index, HbA1c: glycated haemoglobin, ISCED: International Standard Classification of Education, mDM: mitochondrial diabetes mellitus, NMDAS: Newcastle Mitochondrial Disease Adult Scale, ns: not significant, SARA: Scale for the assessment and rating of ataxia.

### Genotypes

**Fig. 2:**
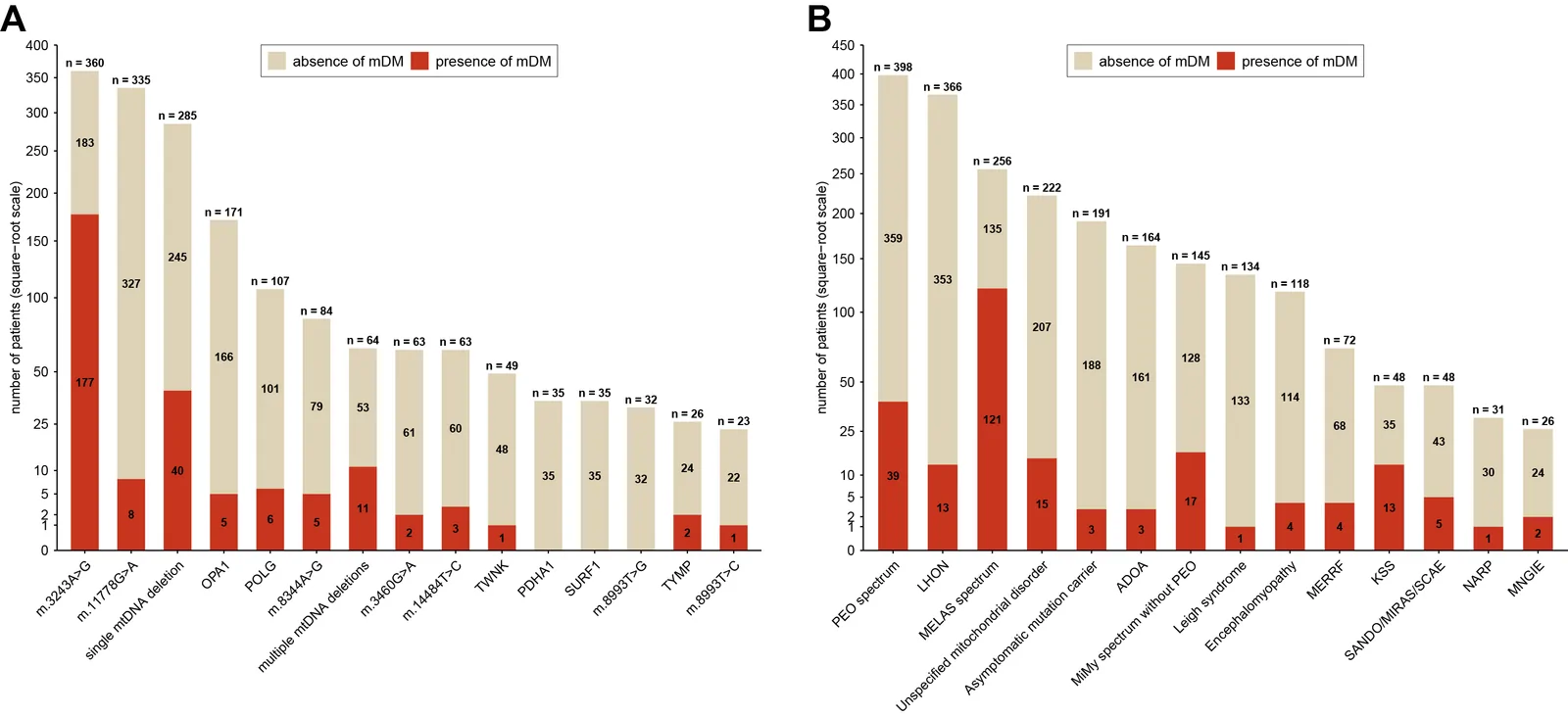
**Prevalence of mitochondrial diabetes mellitus across different genotypes and phenotypes.** Stacked bar chart displaying the absolute number of patients with (red) and without (sand) mDM for A different genotypes and B different phenotypes. Only geno-, and phenotypes with a minimum of ≥20 entries in the registry are shown. Numbers within each bar segment represent absolute patient counts. Total number of patients per genotype group is indicated above each bar (n). The y-axis is displayed on a square-root scale to allow simultaneous visualisation of both large and small genotype groups. Abbreviations: mDM: mitochondrial diabetes mellitus.

**Fig. 3:**
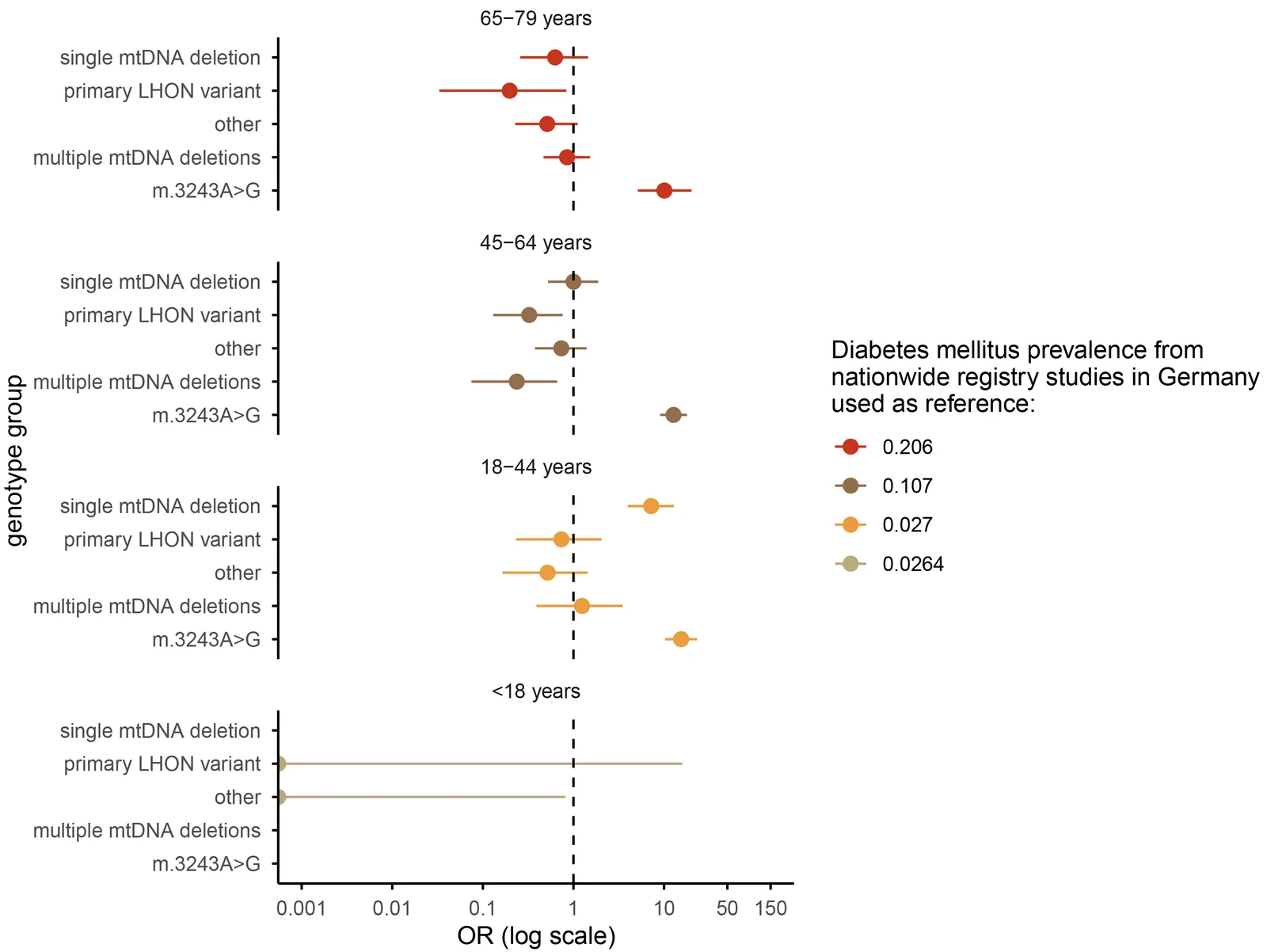
**Age-group dependent prevalence of mitochondrial diabetes mellitus compared to the normal population of Germany.** Forest plot displaying age-, and genotype-dependent OR and respective 95% CI for the risk of having mDM compared to the normal population in Germany. The dashed vertical line at OR = 1 denotes the null effect (no difference from the reference prevalence). Reference prevalence values were derived from nationwide diabetes mellitus registry studies in Germany, stratified by age group (specific values given in the legend). 95% CI for the observed proportions were calculated using the Wilson score method with continuity correction (prop.test, R), and were converted to odds; these were then divided by the reference odds to obtain an odds ratio and its 95% CI relative to the general population reference prevalence. Dots display OR and horizontal line 95% CI. Abbreviations: CI: confidence interval, mDM: mitochondrial diabetes mellitus, OR: odds ratio.

We identified 31 distinct genotypes associated with mDM (eTable 3). For genotypes with ≥20 total registry entries, mDM prevalence is shown in Fig. 2A. The highest frequency occurred in carriers of the m.3243A>G variant (177/360 [49%]). Compared to the normal population in Germany, patients with the m.3243A>G variant had higher odds of having mDM in each age group between 18 and 79 years, see Fig. 3.

### Phenotypes and clinical presentation

mDM prevalence for phenotypes with ≥20 entries is shown in Fig. 2B; we excluded the phenotype MIDD, as this, by definition, includes the presence of mDM. mDM was isolated (without additional symptoms) in 2 cases; most affected individuals had multisystem involvement, commonly neurologic, musculoskeletal, otologic/voice, and ophthalmologic features (eFig. 1). The mean interval between MD onset and mDM onset was 8.5 years, varying by genotype from 7.5 years in the m.3243A>G to 13.2 years in the multiple mtDNA deletions group (eFig. 2).

### Age at onset

**Fig. 4:**
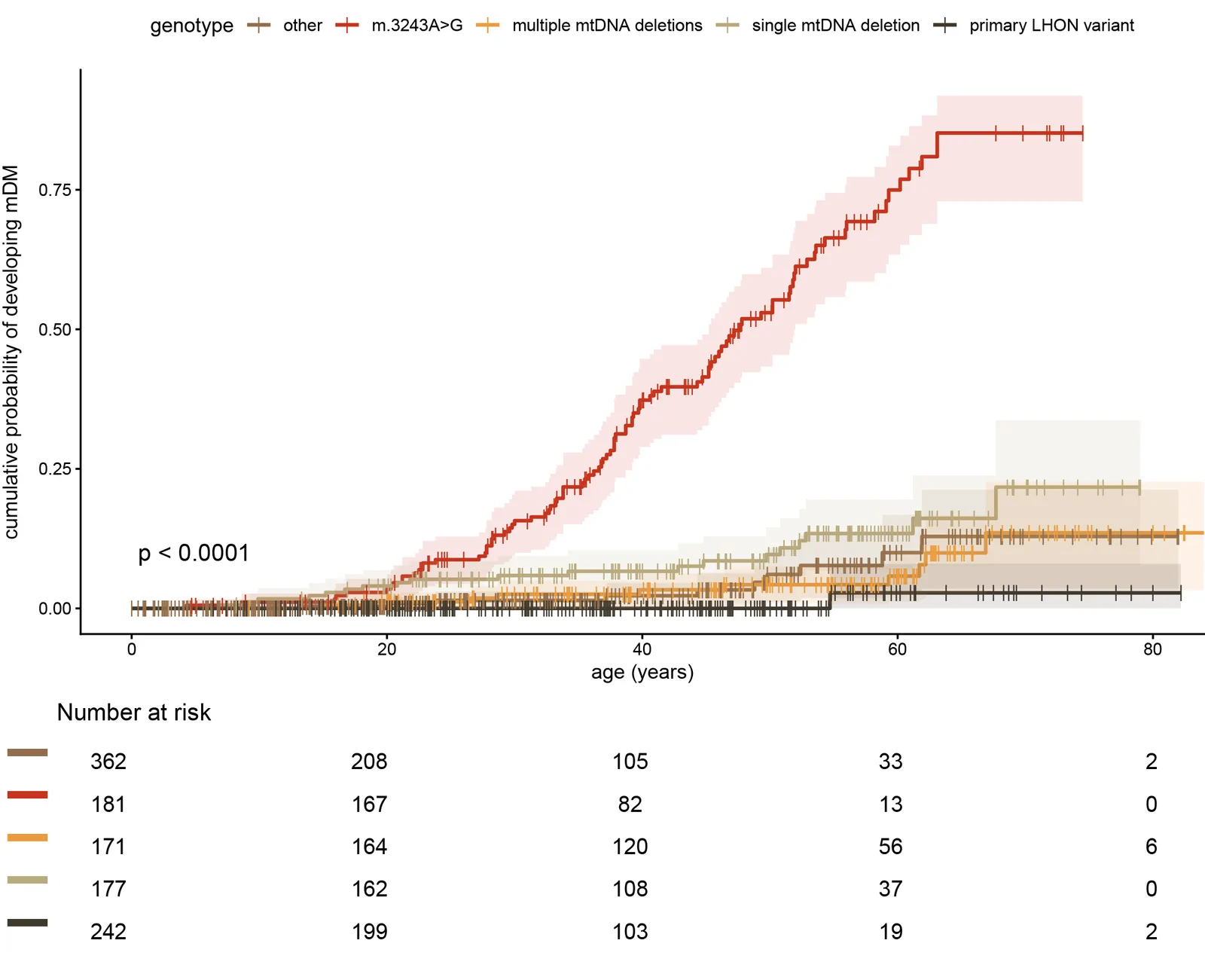
**Kaplan–Meier curve for age at onset of mitochondrial diabetes mellitus.** Kaplan–Meier curve showing cumulative incidence of mDM with age stratified by genotype groups. Survival curves are plotted on the age scale, with the y-axis showing cumulative probability of diabetes onset. Shadowed areas represent 95% CI for survival curves. The log-rank test indicates a significant difference between genotype groups (X^2^ = 324, df = 4; log-rank test, p < 0.0001). Median age at mDM onset was 47.7 years (IQR, 45.2–52) for the m.3243A>G group; in all other genotype groups more than 50% of patients remained mDM-free throughout the study period. Number at risk is shown below each panel at 20, 40, 60, and 80 years of age. Abbreviations: CI: confidence interval, mDM: mitochondrial diabetes mellitus.

Age at onset ranged from 4.0 years to 67.7 years. Kaplan–Meier analysis and Log-rank test showed a significant difference in time to mDM onset by genotype groups (X^2^ = 324, df = 4; Log-rank test, p < 0.0001), with a markedly increased risk within the m.3243A>G group, see Fig. 4. In this group, the median age of mDM onset was 47.7 years (IQR, 45.2–52). For the other groups, the median time to onset could not be estimated as more than 50% of participants remained mDM-free throughout the study period.

### Risk factors for developing mDM

**Fig. 5:**
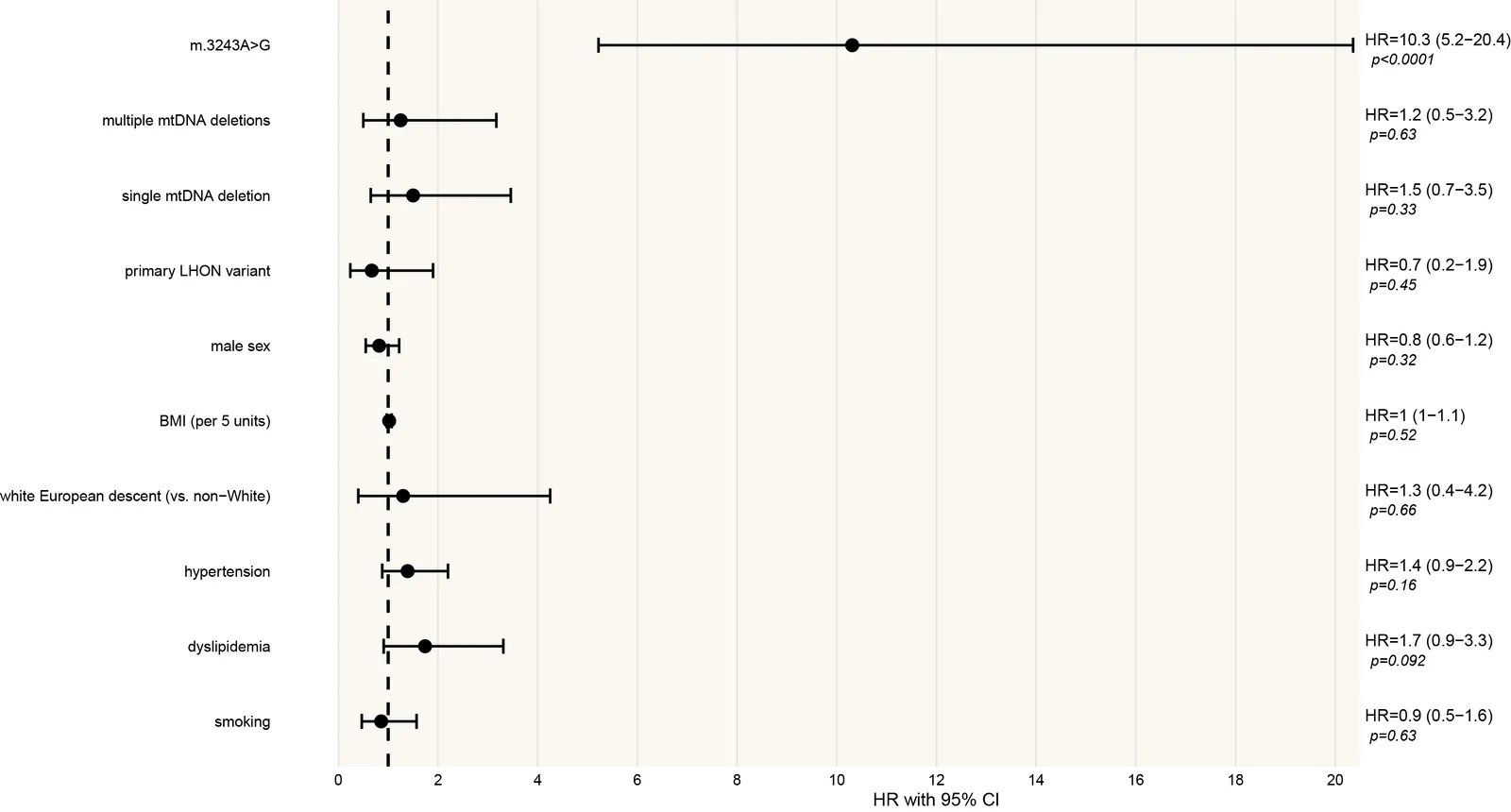
**Cox-proportional Hazards model on the occurrence of mitochondrial diabetes mellitus.** Cox proportional hazards model assessing the influence of genotypes on the occurrence of mDM, adjusted for sex, BMI, race, hypertension, dyslipidaemia, and smoking. Dots within the forest plot represent HR and horizontal lines 95% CI. Vertical dashed line represents baseline (OR = 1). The m.3243A>G genotype was associated with a markedly increased hazard of diabetes onset (HR = 10.3; 95% CI, 5.2–20.4; Cox proportional hazards model, p < 0.0001), while the other genotypes showed no increased risk. Abbreviations: CI: confidence interval, HR: hazard ratio, mDM: mitochondrial diabetes mellitus.

In a Cox proportional hazards model adjusted for sex, BMI, race, hypertension, dyslipidaemia, and smoking, patients within the genotype group m.3243A>G had a higher hazard (HR = 10.3; 95% CI, 5.2–20.4; Cox proportional hazards model, p < 0.0001) of developing mDM compared to those with “other” genotypes (reference), see Fig. 5. The genotype groups multiple mtDNA deletions, single mtDNA deletion, and primary LHON variants as well as sex, BMI, ethnicity, smoking, and the presence of hypertension and dyslipidaemia did not show an increased hazard of developing mDM. The proportional hazards assumption held for all covariates across all imputed datasets (Schoenfeld residual p-values 0.07–0.95). No evidence of multicollinearity (GVIFˆ(1/2df) < 1.06) or influential observations was observed, and the events-per-variable ratio was adequate (11.2). BMI showed apparent non-linearity (p = 0.0097), driven by sparse extreme values; after excluding the outer 1% of the BMI distribution, linearity was supported (p = 0.118), and BMI was retained as a linear term in the final model.

In order to assess risk factors that predispose patients to develop mDM given a specific genotype, we performed multivariable logistic regression within the respective genotype groups, see eFigs. 3 and eTable 4. In the group of the patients with the m.3243A>G mtDNA variant, beside age (OR 2.18; 95% CI, 1.59–2.99; multivariable logistic regression, p < 0.0001), the age-corrected blood heteroplasmy level increased the odds of having mDM significantly (OR: 1.53; 95% CI, 1.21–1.94; multivariable logistic regression, p = 0.0005). Within the multiple mtDNA deletions group, only age was associated with a significantly increased odds of having mDM (OR: 1.54; 95% CI 1.18–2.02; multivariable logistic regression, p = 0.002). In the single mtDNA deletion group, dyslipidaemia was significantly associated with mDM in the multiply imputed dataset (OR: 3.81; 95% CI, 1.12–12.99; multivariable logistic regression, p = 0.033) but not in complete-case analysis (multivariable logistic regression, p = 0.18). Sex, BMI, race, hypertension, and smoking did not show any significant associations with mDM in any model. Minor non-linearity was detected for heteroplasmy in the m.3243A>G model and age in the single mtDNA deletion model; however, linear terms were retained as reasonable approximations given the biological plausibility of a continuous dose-response relationship and the interpretability of odds ratios. All models, except for the m.3243A>G regression, had EPV below 10 and should be interpreted as exploratory. The results for imputed versus non-imputed datasets are given in eTable 5.

### Prevalence of polyneuropathy, retinopathy, and nephropathy

Polyneuropathy, retinopathy, and nephropathy are recognised DM complications but occur also within the phenotypic spectrum of MD. To assess if these conditions occur mDM-related we restricted comparisons to the genotype group m.3243A>G to minimise phenotypic bias. Within this group, data on potential complications (polyneuropathy, retinopathy, and nephropathy) was available for n = 344 patients (n = 177 with mDM, n = 167 without mDM). Polyneuropathy and retinopathy were significantly more frequent among patients with mDM, whereas nephropathy did not differ significantly between groups (eFig. 4). Further, we performed a logistic regression analysis to see if this association remains significant after controlling for covariates. Indeed, the presence of mDM was significantly associated with the occurrence of complications (OR: 3.72; 95% CI, 1.64–8.44; multivariable logistic regression, p = 0.0019). The model was controlled for sex, age, BMI, dyslipidaemia, hypertension, and the age-corrected heteroplasmy level in blood (eTable 6). To examine temporality, we plotted ages at onset of mDM and of each complication across all patients with available data (genotype independent) (eFig. 5).

### Cardiovascular risk factors

T2DM commonly co-occurs with hypertension and dyslipidaemia. We were therefore interested how these conditions are associated with mDM. In our cohort, data on hypertension and dyslipidaemia was available for n = 2220 patients. Both, hypertension and dyslipidaemia, were more prevalent among patients with mDM than among those without mDM (eFig. 6) but did not show a significant association with mDM in the total cohort in the Cox Proportional Hazards model. When only analysing the patients with the m.3243A>G genotype (to control for phenotypic biases), only hypertension but not dyslipidaemia was more prevalent among patients with mDM compared to those without mDM (OR: 8.58; 95% CI, 1.91–79.46; Fisher’s exact test, p = 0.0018). We further performed a logistic regression analysis to see if this association remains significant after controlling for covariates. Indeed, the presence of mDM was significantly associated with the occurrence of hypertension (OR: 5.42; 95% CI, 1.08–27.21; multivariable logistic regression, p = 0.040) after controlling for sex, age, BMI, dyslipidaemia/hypertension respectively, and the age-corrected heteroplasmy level in blood; no significant association was found with dyslipidaemia (eTables 7 and 8).

### Antidiabetic therapies

**Fig. 6:**
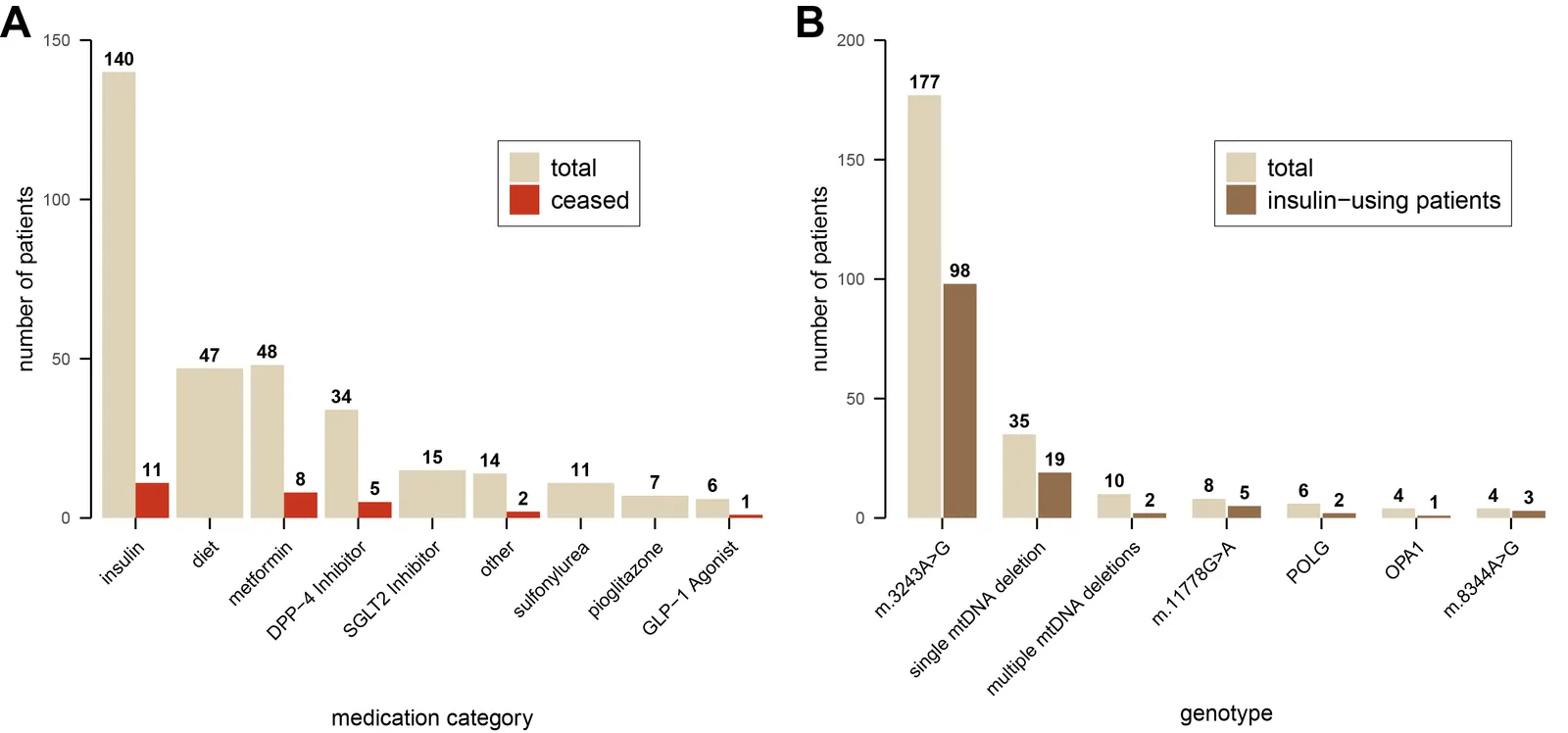
**Antidiabetic therapy regimens in patients with mitochondrial diabetes mellitus.** Bar plot indicating the antidiabetic therapies in the total mDM cohort A, and the number of insulin-dependent patients stratified by genotype B. A In total (sand) 140 (50%) patients with mDM used insulin, and 111 (40%) took oral medications, or injected subcutaneous non-insulin antihyperglycemic agents. Metformin was the most frequently used oral drug; 8 patients discontinued. Additional discontinuations occurred with DPP-4 inhibitors (5 of 34 users) and GLP-1 receptor agonists (1 of 6 patients); discontinuation numbers are displayed in red. B The number of patients using insulin (dark brown) varied between the different genotype groups and was highest in patients with the m.3243A>G variant. Total number of patients with mDM per genotype group are given in sand. Abbreviations: DPP4: dipeptidyl peptidase 4, GLP-1: glucagon-like peptide 1, mitochondrial diabetes mellitus.

Among 281 patients with mDM, 140 (50%) used insulin, 111 (40%) took oral medications or injected subcutaneous non-insulin antihyperglycemic agents, where from 42 (15%) took both (Fig. 6A). Dietary regimens were documented for 47 patients (in 20 as the sole therapy). Nine patients (3%) reported none of the listed therapies. Among oral and subcutaneous non-insulin agents, metformin was most frequently used (n = 48; dose range, 500–2550 mg/d); 8 patients discontinued (1 due to adverse effects; 7 for unspecified reasons). Additional discontinuations occurred with dipeptidyl peptidase 4 (DPP-4) inhibitors (5 of 34 users; reasons not provided) and glucagon-like peptide 1 (GLP-1) receptor agonists (1 of 6; reason not provided). Pioglitazone use was reported only in Italy. The number of patients treated with insulin varied by genotype (Fig. 6B). The mean age at first insulin use was 27.8 years (SD, 14.7; range, 6.9–53.0). Among 15 patients with available dates, the median time from mDM diagnosis to insulin initiation was 0.85 years (IQR, 0.03–2.3). Regarding glycaemic control, the overall longitudinal HbA1c trajectory remained relatively stable over time, ranging from 6.9% to 7.5%, see eFig. 7. No episodes of hypoglycaemia have been reported in the registry, but might, however, reflect a limitation in the data collection process, rather than a true absence of events.

### Literature review of antidiabetic therapies in patients with MD/mDM

We identified 27 publications reporting antihyperglycemic therapy in patients with mDM/MD (eFig. 8). Most data concerned metformin. In 7 reports encompassing 13 individuals with the m.3243A>G variant, neurologic events–often stroke-like episodes–were temporally associated with metformin exposure. By contrast, other publications described 14 patients with mDM with apparently safe metformin use, in some cases for decades, without reported adverse effects. Details are summarised in eTable 9.

## Discussion

In this registry-based analysis of 2399 individuals with genetically confirmed MD, mDM was common, affecting approximately 12% of patients. Carriers of the m.3243A>G variant were most frequently affected, with about half meeting criteria for mDM. Compared to the general population (with an estimated overall prevalence of 0.3–0.4% for T1DM,^20^ and 7–10% for T2DM^21^), DM prevalence was higher in our cohort, indicating an over-average predisposition of patients with MD for DM. This was especially true for distinct genotypes, such as the m.3243A>G variant, where mDM prevalence exceeded the DM risk of the age-matched normal population in every age group. Across 31 genotypes associated with mDM, DM typically occurred as part of a multisystem phenotype rather than as an isolated endocrine disorder.

Historically, mDM has been labelled “type 1–like” or “type 2–like” based on the severity of insulinopenia.^8^ Our data, in line with contemporary understanding, support mDM as a distinct entity of DM. The median age of mDM onset was 47.7 years (45.2–52) in the m.3243A>G group, and diagnosis was made approximately 8.5 years after MD onset, a pattern distinct from both T1DM (early adolescence) and T2DM (peak in the mid-50s to early 70s).^22^^,^^23^ Treatment needs likewise differed: roughly 50% of patients in our cohort were treated with insulin, compared with nearly universal insulin use in T1DM and approximately one quarter in T2DM.^24^ Among those who initiated insulin, transition was rapid (Median [IQR], 0.85 years [0.03–2.3]) from diagnosis in a subset with available dates), consistent with a prominent component of β-cell secretory failure,^25^ with additional contributions from insulin resistance as a pathophysiologic equivalent.^26^ Cardiometabolic comorbidities were frequent in our cohort, yet in patients with the m.3243A>G genotype the presence of mDM was only significantly associated with an increased probability of hypertension, but not dyslipidaemia. Thus, in contrast to T2DM, mDM does not appear to be associated with classic cardiovascular comorbidities in terms of a full-scale metabolic syndrome. Microvascular complications, in particular polyneuropathy and retinopathy, occurred more frequently in patients with mDM than in genotype-matched controls, even after accounting for covariates. In a large analysis of insurance data in Germany, polyneuropathy, retinopathy, and nephropathy were apparent in 14%, 7%, and 8% of patients with T1DM/T2DM, respectively.^27^ Thus, mDM appears to be associated with a similarly or even more severe disease course than classical forms of DM, placing patients at risk for microvascular complications. Selecting antihyperglycemic therapy in MD remains challenging. Prior reports highlight difficulties in maintaining long-term glycaemic control, whereas our cohort demonstrated effective management with a median HbA1c of 6.2% and longitudinal values ranging from 6.9–7.5% on average. Concerns persist regarding metformin, the globally most frequently prescribed oral drug in DM,^28^ because of complex I inhibition and theoretical or reported lactic acidosis risk in mitochondrial disorders.^29^ Our literature review found both case reports of neurologic events temporally associated with metformin in patients with m.3243A>G, and reports of uneventful, long-term metformin use–including over decades–in others. In a recent Delphi consensus,^30^ metformin was considered safe to be used in patients with MD. In our cohort, 8 of 48 metformin users discontinued therapy, of whom only one patient reported side effects, suggesting acceptable tolerability in the vast majority. Nonetheless, the usage of metformin in patients with MD is still controversial, and some product labelling explicitly advises against the use of metformin in this cohort.^31^ Further, alternatives such as DPP-4 inhibitors and GLP-1 receptor agonists have been used in MD/mDM with favourable anecdotal experience.^14^ Prospective studies are needed to define comparative effectiveness and safety.

This study has several limitations. First, its reliance on registry entries introduces risks of misclassification, missing data, and residual confounding; some rare genotypes were sparsely represented. Due to the underrepresentation of several ethnicities, our findings lack generalisability beyond white European descents. Furthermore, due to the absence of information on patient ascertainment (whether participants were probands or family members), our data may be subject to potential ascertainment bias. Second, time-to-event data (e.g., time to insulin initiation) were available only for subsets, limiting precision. Third, islet autoantibodies were not systematically assessed; however, prior work found no islet autoantibodies in carriers of the m.3243A>G variant who were insulin-dependent.^9^ Further, although we distinguished mDM from T2DM at the cohort level, overlap is possible in individual patients–particularly in those who are overweight and non-insulin dependent–underscoring the need for careful clinical adjudication.

In conclusion, mDM can be defined as DM occurring in association with a genetically confirmed MD and not explained by other DM entities. mDM prevalence is high, especially in association with distinct genotypes. There is a wide range in age of onset, spanning from early childhood to late adulthood. Pathophysiology seems to be based on both impaired insulin secretory capacity and, at least to some extent, insulin resistance. Regarding treatment, insulin therapy is more common than oral and other, non-insulin subcutaneous antidiabetic drugs, and the majority of patients on metformin have an uneventful treatment course. Nevertheless, prospective genotype-informed investigations are warranted to facilitate evidence-based, personalised management strategies.

## Contributors

LS, BB, CK, MD, SW, CLM, SS, PF, AM, MS, HP, MM, CL, TK contributed to data acquisition. LS and TK conceptualised and designed the study and wrote the manuscript. LS performed the statistical analysis. LS and TK have accessed and verified the underlying data. BB gave administrative, statistical and material support. BB, CK, MD, SW, CLM, SS, PF, AM, MS, HP, MM, CL critically reviewed the manuscript and gave important intellectual content. All authors read and approved the final version of the manuscript. Members of the GENOMIT study group contributed to data acquisition.

## Data sharing statement

The anonymised data can be accessed on reasonable request addressed to the corresponding author. All analyses were performed with the open-source statistical software R studio. Relevant packages were mice for imputation of data (https://cran.r-project.org/web/packages/mice/index.html), glm for logistic regressions (https://cran.r-project.org/web/packages/glm.predict/index.html), and survival package for Kaplan–Meier curve and Cox proportional hazard model (https://cran.r-project.org/web/packages/survival/index.html).

## Declaration of interests

LS received support for travel by EURO-NMD and European Neuromuscular Centre (ENMC), and honoraria from the German Society for Muscular Diseases (DGM) for lectures. CK, MM and TK are members of the European Reference Network for Neuromuscular Diseases (EURO-NMD). TK is also a member of the European Reference Network for Rare Neurological Diseases (ERN-RND). CK received consulting fees by Symptoma, honoraria by Chiesi and UCB, travel support by Khondrion, and participated on an advisory board of Chiesi and was the co-chair of the mitochondrial working group of the ERN EURO-NMD. SW received travel support by Nutricia Metabolics. CLM received consulting fees, honoraria, travel grants from Chiesi Farmaceutici and Gensight Biologics and participated on an advisory board for Gensight Biologics. SS received support for travel from ERN-EURO-NMD, WCN, and AIM. AM received honoraria by Amius Therapeutics, Sanofi, Argenx, and Hormosan for lectures, and participated on advisory boards of Sanofi and Hormosan receiving personal payments. MS received grants from the German Research Foundation (DFG), AFM/Téléthon, and BMFTR, consulting fees from Atheneum, GLG, Dexter, honoraria and travel grant from UCB, and participated on a Data safety monitoring board from Khondrion and MitoHelp. HP received honoraria from UCB for lectures. TK reports unrelated institutional funding from BMFTR, the German Ministry of Health (BMG), the German Research Foundation (DFG) as well as unrelated institutional funding from pharmaceutical companies Kedrion S.p.A., Khondrion B.V., Leadiant GmbH, OMEICOS Therapeutics GmbH and UCB Biopharma SRL. TK also reports unrelated consulting, advisory board and speaker honoraria from Biogen GmbH, Chiesi GmbH, PharmaLex GmbH, Pretzel Therapeutics Inc., and UCB Biopharma SRL. TK also reports owning stocks of Bayer AG and BioNTech SE. BB, MD, PF, and CL have nothing to declare.

## Appendix A Supplementary data

eFigs. 1–8 and eTables 1–9
