# Supplementary material for: Mitochondrial diabetes mellitus: real world insights from the GENOMIT registry–a multinational, longitudinal cohort study
Source: eBioMedicine. 2026 Sep 1;131:106458. doi: 10.1016/j.ebiom.2026.106458 (PMC13571113; doi:10.1016/j.ebiom.2026.106458)
Supplement: Supplementary file 1 [file mmc1.pdf]

**A Supplementary Materials**

## Mitochondrial Diabetes Mellitus: Real World Insights from the GENOMIT Registry
**-** a multinational, longitudinal cohort study.

Luisa Semmler, MD[1,2,3], Boriana Büchner, MD[4], Cornelia Kornblum, MD[5], Marcus Deschauer, MD[1], Saskia B. Wortmann, MD[6], Chiara La Morgia, MD[7,8], Serenella Servidei, MD[9], Peter Freisinger, MD[10], Alexander Mensch, MD[11,12], Markus Schuelke, MD[13], Holger Prokisch, PhD[2,3,14], Michelangelo Mancuso, MD[15], Costanza Lamperti, MD[16], and Thomas Klopstock, MD[4,17,18]; on behalf of the GENOMIT study group

[1] Department of Neurology, TUM-University Hospital, Munich, Germany
[2] Institute of Human Genetics, TUM-University Hospital, Munich, Germany.
[3] Institute of Neurogenomics, Computational Health Centre, Helmholtz Munich, Neuherberg, Germany.
[4] Friedrich-Baur-Institute at the Department of Neurology, LMU University Hospital, LMU Munich, Munich, Germany
[5] Department of Neuromuscular Diseases, Centre for Neurology, University Hospital Bonn, Bonn, Germany.
[6] University Children's Hospital, Paracelsus Medical University, Salzburg, Austria.
[7] IRCCS Istituto delle Scienze Neurologiche di Bologna, Programma di Neurogenetica, Bologna, Italy.
[8] Department of Biomedical and Neuromotor Sciences, University of Bologna, Bologna, Italy.
[9] Dipartimento di Neuroscienze, Organi di Senso e Torace, Fondazione Policlinico Universitario Agostino Gemelli Irccs, Roma, Italy.
[10] Klinikum am Steinenberg, Children's Hospital Reutlingen, 72764 Reutlingen, Germany.
[11] Department of Neurology, University Medicine Halle, Halle (Saale), Germany.
[12] BG University Hospital Bergmannsheil, Heimer Institute for Muscle Research, Bochum, Germany.
[13] Department of Neuropediatrics, Charité-Universitätsmedizin Berlin, Corporate Member of Freie Universität Berlin, Humboldt-Universität Berlin, and Berlin Institute of Health, Berlin, Germany.
[14] German Centre for Child and Adolescent Health (DZKJ), partner site Munich, Munich, Germany.
[15] Department of Clinical and Experimental Medicine, Neurological Institute, University of Pisa, Pisa, Italy.
[16] UO di Genetica Medica e Neurogenetica, Fondazione IRCCS Istituto Neurologico C. Besta, Milan, Italy.
[17] Munich Cluster for Systems Neurology (SyNergy), Munich, Germany.
[18] German Centre for Neurodegenerative Diseases (DZNE), Munich, Germany.

Corresponding author: Thomas Klopstock, MD; Friedrich-Baur-Institute at the Department of Neurology, LMU University Hospital, LMU Munich, Munich, Germany. phone: +49-89-4400-57400; email: Thomas Klopstock@med.LMU.de

**Table of Contents**

**eTable 1. Study population characteristics in the subgroup of patients with the m.3243A>G genotype** ........ 3
**eTable 2. Differences between Italian and German mDM population** ........ 5
**eTable 3. Distinct genotypes associated with mDM** ........ 7
**eFigure 1. Affected organ systems in mDM patients and their interactions** ........ 9
**eFigure 2. Time between MD onset and mDM onset stratified by genotype** ........ 10
**eFigure 3. Multiple logistic regression analyses on the risk of developing mDM within each genotype group** ........ 11
**eTable 4. Multiple logistic regression analyses on the occurrence of mDM within the respective genotype groups** ........ 12
**eTable 5. Imputed versus non-imputed data** ........ 13
**eFigure 4. Polyneuro-, retino-, and nephropathy in m.3243A>G patients with mDM compared to those without mDM** ........ 15
**eTable 6. Logistic regression on the relevance of mDM status for having complications such as polyneuro-, retino-, and nephropathy** ........ 16
**eFigure 5. Occurrence of mDM and complication diagnosis** ........ 17
**eFigure 6. Prevalence of cardiovascular risk factors dyslipidemia and hypertension in mDM and non-mDM patients** ........ 18
**eTable 7. Logistic regression analysis on the influence of mDM presence on the likelihood of having hypertension in m.3243A>G patients** 19
**eTable 8. Logistic regression analysis on the influence of mDM presence on the likelihood of having dyslipidemia in m.3243A>G patients** . 20
**eFigure 7. Longitudinal HbA1c Trajectories Relative to Baseline Measurement to display long-term glycemic control** ........ 21
**eFigure 8. PRISMA flow chart summarizing the results of the literature search on antidiabetic drug usage in MD patients** ........ 22
**eTable 9. Results of the literature review on antidiabetic drug usage in MD patients** ........ 23
**References** ........ 26

**eTable 1. Study population characteristics in the subgroup of patients with the m.3243A>G genotype**

| Parameter | m.3243A>G mDM | m.3243A>G non mDM | m.3243A>G total cohort | Adjusted p value |
|---|---|---|---|---|
| Count [n] | 177 | 183 | 360 | |
| Age [yr] | 57.4 (47.4-63.8) | 43.1 (29.95-53.25) | 50.8 (37.8-60.8) | <0.0001 |
| Height [cm] | 165 (158-169) | 165 (158-172) | 165 (158-170) | 1.0 |
| Weight [kg] | 55 (48.12-65.88) | 54 (48-65) | 54 (48-65) | 1.0 |
| BMI [kg/m²] | 20.68 (18.7-23.37) | 19.93 (18.03-23.34) | 20.11 (18.23-23.39) | 1.0 |
| HbA1c [%] | 7.1 (6.5-7.2) | 5.3 (5.1-5.5) | 6.5 (5.62-7.1) | <0.0001 |
| NMDAS [points] | 19 (12-28) | 11 (5-18.5) | 15 (10-26) | <0.0001 |
| SARA [points] | 3 (1-8) | 2 (0-5.12) | 2 (0-7) | 1.0 |
| Heteroplasmy in blood of m.3243A>G diabetes [%] | 60.02 (44.86-97.99) | 46.19 (22.7-86.91) | 57.53 (30.28-90.48) | 0.062 |
| Age of PMD onset [years] | 27.8 (19.7-41.1) | 23.3 (13.4-37.2) | 25.3 (16.85-39.73) | 0.027 |
| Time of follow-up [years] | 3.27 (1.6-6.04) | 2.8 (1.52-4.61) | 3.01 (1.54-5.6) | 1.0 |
| Deaths [n (%)] | 15 (8.5) | 12 (6.6) | 27 (7.5) | 1.0 |
| Mortality Rate [Deaths per 1,000 person years] | 33.8 | 39.93 | 36.27 | |
| Sex [n (%)] | | | | 0.44 |
| female | 117 (66.1%) | 113 (61.7%) | 230 (63.9%) | |
| male | 60 (33.9%) | 70 (38.3%) | 130 (36.1%) | |
| Race [n (%)] | | | | 0.52 |
| White European descent | 147 (96.1%) | 142 (94.7%) | 289 (95.4%) | |
| other | 0 (0%) | 1 (0.7%) | 1 (0.3%) | |
| Asian (other) | 1 (0.7%) | 3 (2.0%) | 4 (1.3%) | |
| Multi-racial | 1 (0.7%) | 2 (1.3%) | 3 (1.0%) | |
| Hispanic | 1 (0.7%) | 0 (0%) | 1 (0.3%) | |
| Native American | 2 (1.3%) | 0 (0%) | 2 (0.7%) | |
| Black (African) | 1 (0.7%) | 2 (1.3%) | 3 (1.0%) | |
| Education [n (%)] | | | | 0.07 |
| ISCED 1 - primary education | 0 (0%) | 4 (5.4%) | 4 (2.8%) | |
| ICSED 2 - lower seconday education | 10 (14.1%) | 10 (13.5%) | 20 (13.8%) | |
| ISCED 3 - upper secondary education | 23 (32.4%) | 24 (32.4%) | 47 (32.4%) | |
| ISCED 4 - post-secondary non-tertiary education | 13 (18.3%) | 10 (13.5%) | 23 (15.9%) | |
| ISCED 5 - short-cycle tertiary education | 5 (7.0%) | 6 (8.1%) | 11 (7.6%) | |
| ISCED 6 - Bachelor or equivalent | 18 (25.4%) | 9 (12.2%) | 27 (18.6%) | |
| ISCED 7 - Master or equivalent | 1 (1.4%) | 5 (6.8%) | 6 (4.1%) | |
| ISCED 8 - Doctoral or equivalent | 1 (1.4%) | 2 (2.7%) | 3 (2.1%) | |
| ISCED 9 - not elsewhere classified | 0 (0%) | 0 (0%) | 0 (0%) | |
| Diet [n (%)] | | | | 0.02 |
| no diet (normal nutrition) | 63 (72.4%) | 70 (89.7%) | 133 (80.6%) | |
| other | 5 (5.7%) | 1 (1.3%) | 6 (3.6%) | |
| vegetarian | 0 (0%) | 0 (0%) | 0 (0%) | |
| mediterranean | 17 (19.5%) | 6 (7.7%) | 23 (13.9%) | |
| low protein | 2 (2.3%) | 0 (0%) | 2 (1.2%) | |

| | | | | |
|---|---|---|---|---|
| low fat | 0 (0%) | 1 (1.3%) | 1 (0.6%) | |
| Smoking [n (%)] | | | | 0.22 |
| no | 97 (85.8%) | 93 (80.9%) | 190 (83.3%) | |
| current smoker | 6 (5.3%) | 14 (12.2%) | 20 (8.8%) | |
| former smoker | 9 (8.0%) | 6 (5.2%) | 15 (6.6%) | |
| occasional smoker | 1 (0.9%) | 2 (1.7%) | 3 (1.3%) | |
| Alcohol [n (%)] | | | | 0.71 |
| no | 72 (83.7%) | 66 (81.5%) | 138 (82.6%) | |
| yes, daily | 1 (1.2%) | 0 (0%) | 1 (0.6%) | |
| yes, weekly | 2 (2.3%) | 4 (4.9%) | 6 (3.6%) | |
| yes, occasionally | 11 (12.8%) | 11 (13.6%) | 22 (13.2%) | |

Study population characteristics in the subgroup of patients with the m.3243A>G genotype, stratified by mDM status. We excluded n = 17 patients with impaired glucose tolerance (IGT) and/or transient mDM to avoid misclassification bias in both the mDM and control groups. Continuous variables are presented as median (IQR) and categorical variables as number (%). Differences between groups were assessed using the Wilcoxon rank-sum (Mann-Whitney) test for continuous variables and Fisher's exact test for categorical variables. Patients with the m.3243A>G genotype and mDM were significantly older, had a higher HbA1c, and scored higher on the NMDAS compared to those with the m.3243A>G genotype without mDM. Levels of education and diet also differed significantly between the two groups. Abbreviations: BMI: body mass index, HbA1c: glycated haemoglobin, ISCED: International standard classification of education, mDM: mitochondrial diabetes mellitus, NMDAS: Newcastle Mitochondrial Disease Adult Scale, SARA: Scale for the Assessment and Rating of Ataxia

## eTable 2. Differences between Italian and German mDM population

| Parameter | Italian | German | Adjusted p value |
|---|---|---|---|
| Count [n] | 160 | 121 | |
| Age [yr] | 58.2 (47.83-67.58) | 57.7 (45.7-67.2) | 1.0 |
| Height [cm] | 165 (158-169.5) | 168 (161-173.25) | 0.034 |
| Weight [kg] | 54 (49-65.5) | 65 (53.72-79.25) | 0.00077 |
| BMI [kg/m²] | 21.09 (18.73-23.48) | 23.02 (19.25-27.82) | 0.042 |
| Underweight (BMI < 18.5 kg/m$^2$) [n (%)] | 20 (20 %) | 21 (18%) | |
| Normal (BMI: 18.5-24.9 kg/m$^2$) | 64 (65%) | 53 (44%) | |
| Overweight (BMI: 25.0-29.9 kg/m$^2$) | 11 (11%) | 25 (21%) | |
| Obesity (BMI ≥ 30 kg/m$^2$) | 4 (4%) | 21 (18%) | |
| HbA1c [%] | NA (NA-NA) | 6.95 (6.5-7.35) | NA |
| NMDAS [points] | 19.5 (12-28.25) | 18 (12-30) | 1.0 |
| SARA [points] | 6.5 (4.75-10) | 2 (0.12-7) | 1.0 |
| Heteroplasmy in blood of m.3243A>G diabetes [%] | 55.17 (42.1-80.48) | 75.95 (50.2-113.33) | 1.0 |
| Age of PMD onset [years] | 28.05 (19.4-45.27) | 27.8 (16.3-46.95) | 1.0 |
| Time of follow-up [years] | 3.37 (1.53-4.63) | 3.25 (1.89-6.72) | 1.0 |
| Deaths [n (%)] | 16 (10) | 4 (3.3) | 0.34 |
| Mortality Rate [Deaths per 1,000 person years] | 49.7 | 11.61 | |
| Sex [n (%)] | | | 0.46 |
| female | 101 (63.1%) | 71 (58.7%) | |
| male | 59 (36.9%) | 50 (41.3%) | |
| Race [n (%)] | | | 0.032 |
| White European descent | 125 (96.2%) | 115 (95.8%) | |
| other | 0 (0%) | 1 (0.8%) | |
| Asian (other) | 0 (0%) | 3 (2.5%) | |
| Multi-racial | 0 (0%) | 1 (0.8%) | |
| Hispanic | 1 (0.8%) | 0 (0%) | |
| Native American | 3 (2.3%) | 0 (0%) | |
| Black (African) | 1 (0.8%) | 0 (0%) | |
| Education [n (%)] | | | 0.0034 |
| ISCED 1 - primary education | 2 (2.9%) | 1 (2.0%) | |
| ICSED 2 - lower seconday education | 14 (20.6%) | 7 (14.3%) | |
| ISCED 3 - upper secondary education | 25 (36.8%) | 9 (18.4%) | |
| ISCED 4 - post-secondary non-tertiary education | 5 (7.4%) | 15 (30.6%) | |
| ISCED 5 - short-cycle tertiary education | 4 (5.9%) | 6 (12.2%) | |
| ISCED 6 - Bachelor or equivalent | 17 (25.0%) | 8 (16.3%) | |
| ISCED 7 - Master or equivalent | 1 (1.5%) | 0 (0%) | |
| ISCED 8 - Doctoral or equivalent | 0 (0%) | 2 (4.1%) | |
| ISCED 9 - not elsewhere classified | 0 (0%) | 1 (2.0%) | |
| Diet [n (%)] | | | 0.0001 |
| no diet (normal nutrition) | 51 (63.0%) | 50 (86.2%) | |

| | | | |
|---|---|---|---|
| other | 3 (3.7%) | 7 (12.1%) | |
| vegetarian | 0 (0%) | 1 (1.7%) | |
| mediterranean | 24 (29.6%) | 0 (0%) | |
| low protein | 2 (2.5%) | 0 (0%) | |
| low fat | 1 (1.2%) | 0 (0%) | |
| Smoking [n (%)] | | | 0.63654 |
| no | 71 (85.5%) | 94 (84.7%) | |
| current smoker | 3 (3.6%) | 8 (7.2%) | |
| former smoker | 7 (8.4%) | 8 (7.2%) | |
| occasional smoker | 2 (2.4%) | 1 (0.9%) | |
| Alcohol [n (%)] | | | 0.0002 |
| no | 75 (94.9%) | 38 (64.4%) | |
| yes, daily | 0 (0%) | 2 (3.4%) | |
| yes, weekly | 0 (0%) | 3 (5.1%) | |
| yes, occasionally | 4 (5.1%) | 16 (27.1%) | |

Descriptive analysis for continuous and categorical variables separately for the Italian, and German study populations. Continuous variables are given in median (IQR), categorical variables in number (%). Differences between the groups were assessed using Wilcoxon rank-sum test (Mann-Whitney) for continuous variables and Fisher exact test. German patients with mDM were significantly taller, had a higher body weight, and an increased BMI compared to the Italian mDM population; further differences occurred in race distribution, education level, dietary regimens, and alcohol consumption. Abbreviations: BMI: body mass index, HbA1c: glycated haemoglobin, ISCED: International standard classification of education, mDM: mitochondrial diabetes mellitus, NMDAS: Newcastle Mitochondrial Disease Adult Scale, SARA: Scale for the Assessment and Rating of Ataxia.

**eTable 3. Distinct genotypes associated with mDM**

| Genotype | Frequency, No. (%) |
|---|---|
| ETFDH | 1 (0.4) |
| m.11778 G>A | 8 (2.9) |
| m.14258G>A; m.14582A>G | 1 (0.4) |
| m.14484 T>C | 3 (1.1) |
| m.14709 T>C | 2 (0.7) |
| m.3243 A>G | 177 (63.0) |
| m.3243 A>T | 1 (0.4) |
| m.3248 G>A | 1 (0.4) |
| m.3255A>G | 1 (0.4) |
| m.3260A>G | 1 (0.4) |
| m.3302 A>G | 1 (0.4) |
| m.3460 G>A | 2 (0.7) |
| m.3697 G>A | 1 (0.4) |
| m.7471insC | 1 (0.4) |
| m.8344 A>G | 5 (1.8) |
| m.8363 G>A | 1 (0.4) |
| m.8364 G>A | 1 (0.4) |
| m.8782 G>A | 1 (0.4) |
| m.8993 T>C | 1 (0.4) |
| m.9049G>A; m.14724G>A | 1 (0.4) |
| m.9176 C>T | 1 (0.4) |
| m.9176T>C | 1 (0.4) |
| m.9334 T>C | 1 (0.4) |
| multiple mtDNA deletions | 11 (3.9) |
| OPA1 | 5 (1.8) |
| POLG | 6 (2.1) |
| single mtDNA deletion | 40 (14.2) |
| SLC25A4 | 1 (0.4) |

| Genotype | Frequency, No. (%) |
|---|---|
| TK2 | 1 (0.4) |
| TWNK | 1 (0.4) |
| TYMP | 2 (0.7) |

**eFigure 1. Affected organ systems in mDM patients and their interactions**

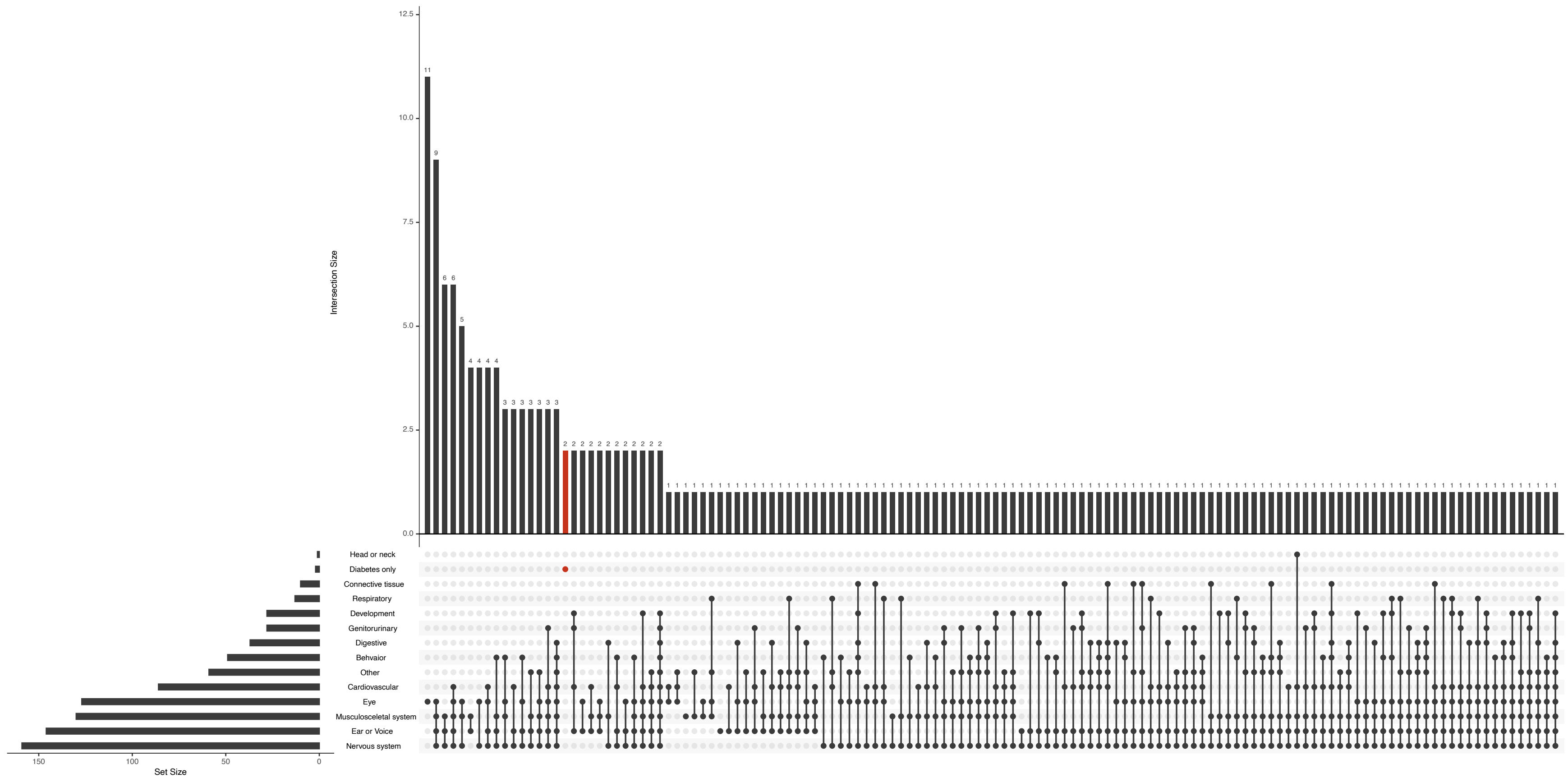


UpSet plot visualizing intersections between different organ involvements of mDM patients. Each column in the matrix represents a unique combination of sets (intersection), with black dots indicating membership. The horizontal bar chart on top shows the number of occurrences of the respective organ involvement intersections; the vertical bar chart on the left shows the total size of each set. Most frequently, large intersections with multiple affected organ systems were present. Only in two patients, diabetes was an isolated symptom without other organs being affected.

**eFigure 2. Time between MD onset and mDM onset stratified by genotype**

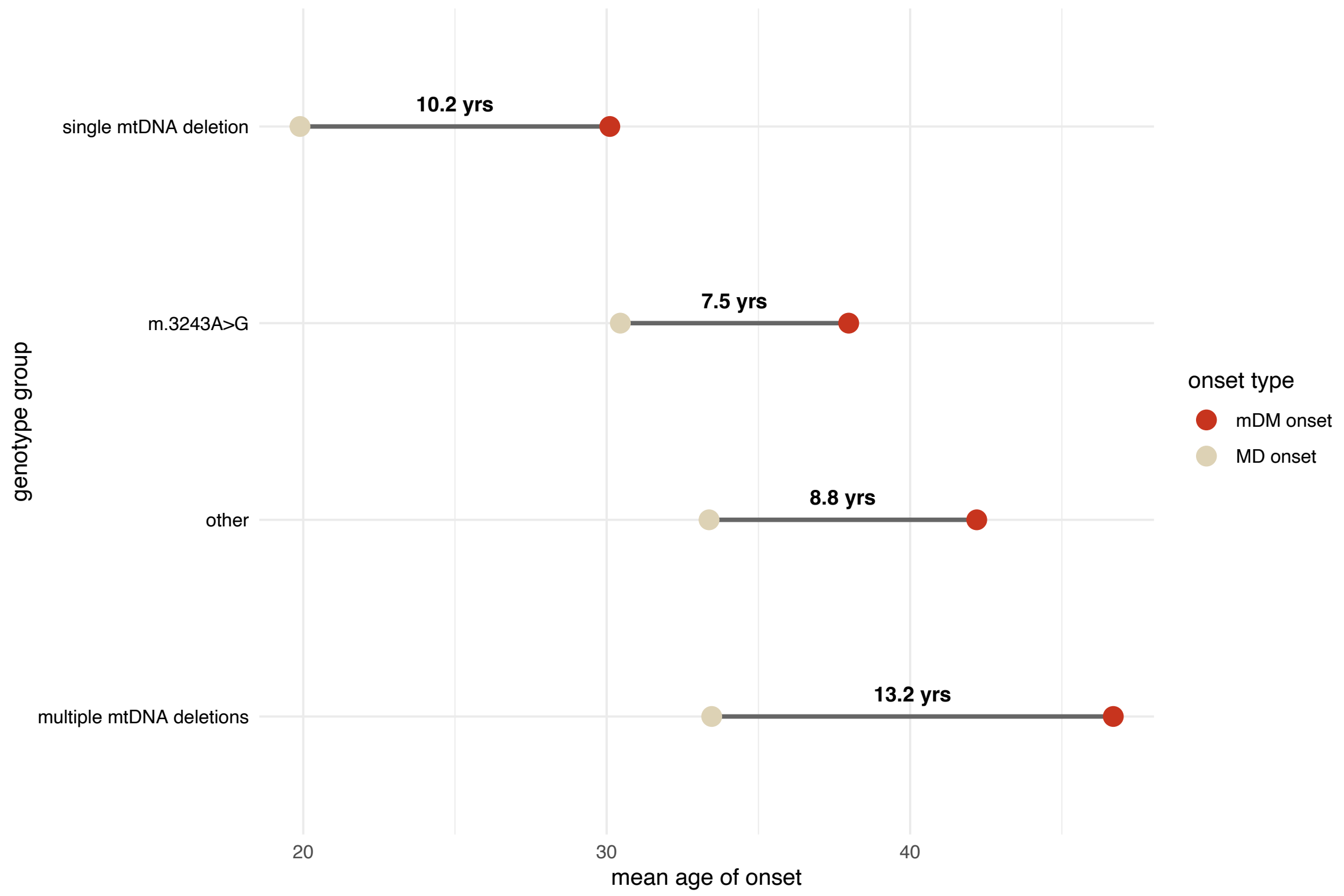


Chart displaying the time difference between mDM onset (red) and MD (sand) onset per genotype. The line between the points indicates the time difference in years; calculations were performed on an individual level and summarized before plotting. mDM occurred on average 8.5 years after the diagnosis of MD. This time varied depending on the genotype from 7.5 years in the m.3243A>G to 13.2 years in the multiple mtDNA deletion group.
mDM: mitochondrial diabetes mellitus, MD: mitochondrial disease.

**eFigure 3. Multiple logistic regression analyses on the risk of developing mDM within each genotype group**

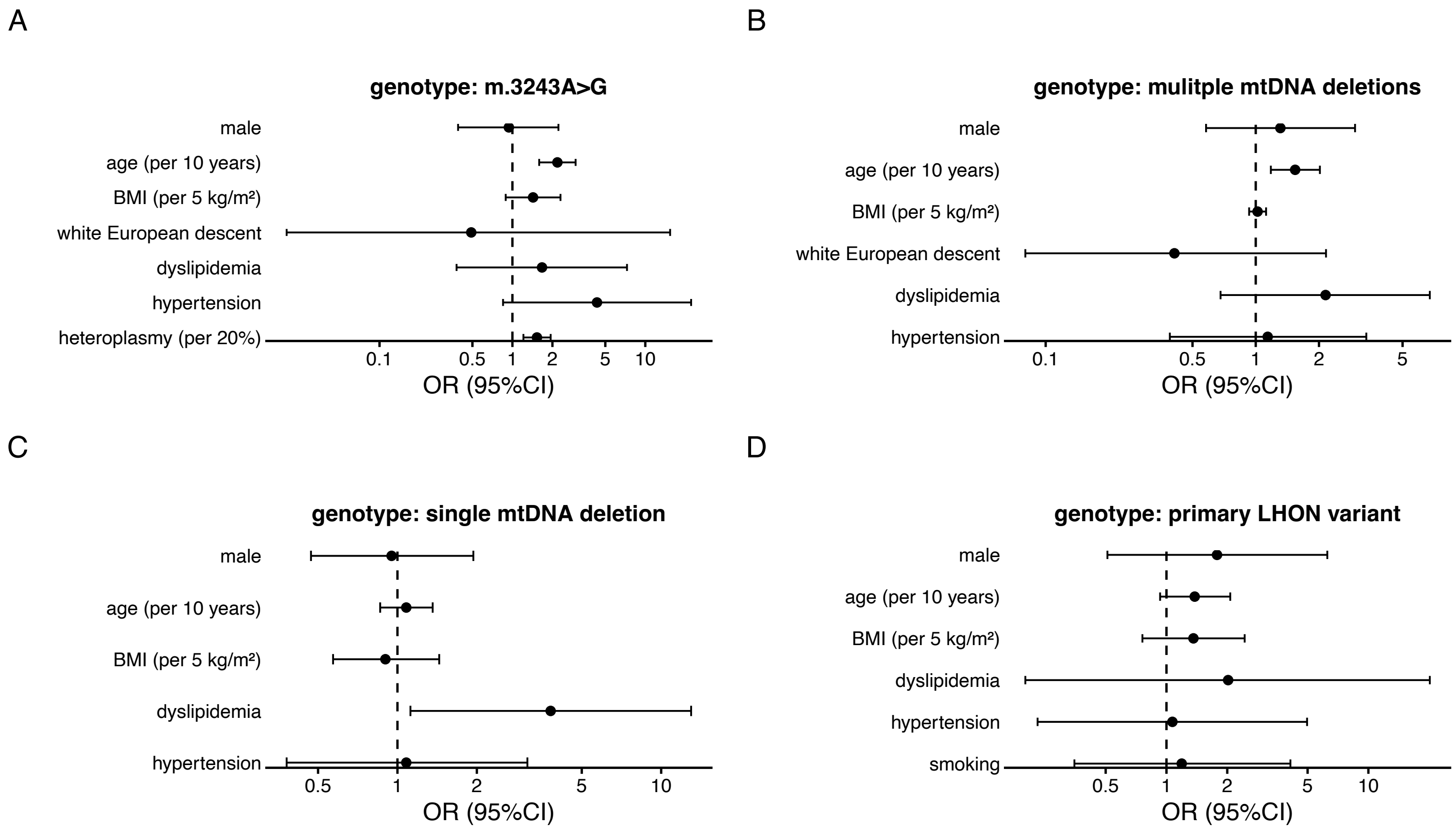


Forest plots of the multivariable logistic regressions performed to assess the association between the onset of mDM and the genotypes A m.3243A>G (n = 176), B multiple mtDNA deletions (n = 392), C single mtDNA deletion (n = 272), and D primary LHON variants (n = 420). The models were adjusted for age, sex, BMI, dyslipidemia, and hypertension; as well as model A for the age-corrected blood heteroplasmy level and model D for smoking. OR with 95% CI are shown for each predictor. Reference categories: genotype = other, sex = female; race = non-white, dyslipidemia = no dyslipidemia, hypertension = no hypertension, smoking = no smoking. Analysis was conducted in R using the glm() function with binomial family on imputed datasets. Abbreviations: CI: confidence interval, mDM: mitochondrial diabetes mellitus, OR: Odds ratio.

**eTable 4. Multiple logistic regression analyses on the occurrence of mDM within the respective genotype groups**

| | **A**<br>**m.3243A>G**<br>Nagelkerle $R^2$ = 0.43, AUC = 0.83,<br>p <0.0001, Hosmer-Lemeshow p=0.30 | | **B**<br>**multiple mtDNA deletions**<br>Nagelkerle $R^2$ = 0.10, AUC = 0.75,<br>p =0.012, Hosmer-Lemeshow p=0.008 | | **C**<br>**Single mtDNA deletion**<br>Nagelkerle $R^2$ = 0.037, AUC = 0.59,<br>p =0.33, Hosmer-Lemeshow p=0.59 | | **D**<br>**Primary LHON variant**<br>Nagelkerle $R^2$ = 0.073, AUC = 0.73,<br>p =0.28, Hosmer-Lemeshow p=0.35 | |
|---|---|---|---|---|---|---|---|---|
| **Parameter** | **Odds ratio (95%CI)** | **P value** | **Odds ratio (95%CI)** | **P value** | **Odds ratio (95%CI)** | **P value** | **Odds ratio (95%CI)** | **P value** |
| Intercept | 0.00 (0.00–0.14) | 0.0038 | 0.01 (0.00–0.07) | <0.0001 | 0.17 (0.03–1.04) | 0.055 | 0.00 (0.00–0.04) | 0.00041 |
| Male | 0.94 (0.39–2.22) | 0.88 | 1.31 (0.58–2.97) | 0.52 | 0.95 (0.47–1.94) | 0.90 | 1.78 (0.51–6.26) | 0.37 |
| Age (per 10 years) | 2.18 (1.59–2.99) | <0.0001 | 1.54 (1.18–2.02) | 0.002 | 1.08 (0.86–1.36) | 0.51 | 1.38 (0.93–2.07) | 0.11 |
| BMI (per 5kg/m2) | 1.43 (0.89–2.30) | 0.14 | 1.02 (0.93–1.12) | 0.63 | 0.90 (0.57–1.44) | 0.66 | 1.36 (0.76–2.43) | 0.3 |
| White European descent | 0.49 (0.02–15.37) | 0.67 | 0.41 (0.08–2.16) | 0.29 | NA | NA | NA | NA |
| Dyslipidemia | 1.67 (0.38–7.28) | 0.49 | 2.15 (0.68–6.74) | 0.19 | 3.81 (1.12–12.99) | 0.033 | 2.02 (0.20–20.15) | 0.55 |
| Hypertension | 4.33 (0.85–22.12) | 0.078 | 1.14 (0.39–3.36) | 0.81 | 1.08 (0.38–3.11) | 0.88 | 1.07 (0.23–4.97) | 0.94 |
| Heteroplasmy (per 20%) | 1.53 (1.21–1.94) | 0.0005 | NA | NA | NA | NA | NA | NA |
| Smoking | NA | NA | NA | NA | NA | NA | 1.19 (0.35–4.11) | 0.78 |

Numeric values of the multivariable logistic regression (forest plots shown in eFigure3) between the onset of mDM and the genotypes A m.3243A>G (n = 176), B multiple mtDNA deletions (n = 392), C single mtDNA deletion (n = 272), and D primary LHON variants (n = 420). The models were adjusted for age, sex, BMI, dyslipidemia, and hypertension; as well as model A for the age-corrected blood heteroplasmy level and model D for smoking. OR with 95% CI are shown for each predictor. P values indicate statistical significance (p<0.05).

## eTable 5. Imputed versus non-imputed data

| **Cox proportional hazard model<br>Full imputed dataset: n = 1162, Complete cases: n = 788** | | | | |
|---|---|---|---|---|
| **Variable** | **HR (95% CI) - Imputed** | **HR (95% CI) - Complete case** | **p - Imputed** | **p - Complete case** |
| m.3243A>G | 10.31 (5.22–20.36) | 10.13 (5.17–19.86) | <0.0001 | <0.0001 |
| multiple mtDNA deletions | 1.25 (0.50–3.17) | 1.23 (0.49–3.08) | 0.63 | 0.66 |
| single mtDNA deletion | 1.50 (0.65–3.46) | 1.48 (0.65–3.37) | 0.33 | 0.35 |
| primary LHON variant | 0.67 (0.24–1.90) | 0.66 (0.24–1.85) | 0.45 | 0.43 |
| Male sex | 0.82 (0.55–1.22) | 0.83 (0.56–1.24) | 0.32 | 0.37 |
| BMI (per 5 kg/m²) | 1.02 (0.97–1.07) | 1.02 (0.97–1.07) | 0.52 | 0.52 |
| White European descent | 1.30 (0.40–4.25) | 1.31 (0.41–4.23) | 0.66 | 0.65 |
| Dyslipidemia | 1.74 (0.91–3.31) | 1.72 (0.91–3.25) | 0.092 | 0.095 |
| Hypertension | 1.39 (0.88–2.20) | 1.39 (0.88–2.19) | 0.16 | 0.15 |
| Smoking | 0.86 (0.47–1.57) | 0.86 (0.47–1.57) | 0.63 | 0.63 |
| **Logistic regression m.3243A>G<br>Full imputed dataset: n = 176, Complete cases: n = 119** | | | | |
| **Variable** | **OR (95% CI) - Imputed** | **OR (95% CI) - Complete case** | **p - Imputed** | **p - Complete case** |
| Male sex | 0.94 (0.39–2.22) | 0.56 (0.20–1.55) | 0.88 | 0.27 |
| Age (per 10 years) | 2.18 (1.59–2.99) | 2.34 (1.60–3.71) | <0.0001 | <0.0001 |
| BMI (per 5 kg/m²) | 1.43 (0.89–2.30) | 1.46 (0.87–2.55) | 0.14 | 0.16 |
| White European descent | 0.49 (0.02–15.37) | 0.40 (0.02–5.46) | 0.67 | 0.49 |
| Dyslipidemia | 1.67 (0.38–7.28) | 5.34 (0.97–42.91) | 0.49 | 0.071 |
| Hypertension | 4.33 (0.85–22.12) | 8.35 (1.25–170.23) | 0.078 | 0.064 |
| Heteroplasmy (per 20%) | 1.53 (1.21–1.94) | 1.58 (1.23–2.15) | 0.00050 | 0.0012 |
| **Logistic regression multiple mtDNA deletions<br>Full imputed dataset: n = 392, Complete cases: n = 223** | | | | |
| **Variable** | **OR (95% CI) - Imputed** | **OR (95% CI) - Complete case** | **p - Imputed** | **p - Complete case** |
| Male sex | 1.31 (0.58–2.97) | 0.97 (0.38–2.44) | 0.52 | 0.95 |
| Age (per 10 years) | 1.54 (1.18–2.02) | 1.43 (1.10–1.91) | 0.0017 | 0.011 |
| BMI (per 5 kg/m²) | 1.02 (0.93–1.12) | 1.00 (NA–1.08) | 0.63 | 0.96 |
| White European descent | 0.41 (0.08–2.16) | 0.46 (0.10–3.30) | 0.29 | 0.36 |
| Dyslipidemia | 2.15 (0.68–6.74) | 3.51 (0.92–12.23) | 0.19 | 0.053 |
| Hypertension | 1.14 (0.39–3.36) | 0.81 (0.21–2.63) | 0.81 | 0.74 |
| **Logistic regression single mtDNA deletion<br>Full imputed dataset: n = 272, Complete cases: n = 223** | | | | |
| **Variable** | **OR (95% CI) - Imputed** | **OR (95% CI) - Complete case** | **p - Imputed** | **p - Complete case** |
| Male sex | 0.95 (0.47–1.94) | 1.13 (0.49–2.53) | 0.90 | 0.77 |
| Age (per 10 years) | 1.08 (0.86–1.36) | 1.27 (0.99–1.65) | 0.51 | 0.068 |
| BMI (per 5 kg/m²) | 0.90 (0.57–1.44) | 0.94 (0.62–1.36) | 0.66 | 0.75 |
| Dyslipidemia | 3.81 (1.12–12.99) | 2.76 (0.54–11.71) | 0.033 | 0.18 |
| Hypertension | 1.08 (0.38–3.11) | 0.50 (0.11–1.71) | 0.88 | 0.32 |

| **Logistic regression primary LHON variant**<br>**Full imputed dataset: n = 420, Complete cases: n = 268** | | | | |
|---|---|---|---|---|
| **Variable** | **OR (95% CI) - Imputed** | **OR (95% CI) - Complete case** | **p - Imputed** | **p - Complete case** |
| Male sex | 1.78 (0.51–6.26) | 2.29 (0.56–11.88) | 0.37 | 0.27 |
| Age (per 10 years) | 1.38 (0.93–2.07) | 1.69 (1.03–2.89) | 0.11 | 0.042 |
| BMI (per 5 kg/m²) | 1.36 (0.76–2.44) | 1.41 (0.75–2.43) | 0.30 | 0.24 |
| Dyslipidemia | 2.02 (0.20–20.15) | 0.00 (NA-3809299037083526738766865435312260848605617936185021072690553353666 56.00) | 0.55 | 0.99 |
| Hypertension | 1.07 (0.23–4.97) | 1.18 (0.20–5.70) | 0.94 | 0.84 |
| Smoking | 1.19 (0.35–4.11) | 1.57 (0.41–6.02) | 0.78 | 0.50 |

Presentation of imputed versus non-imputed datasets used for the Cox-proportional hazards model as well as the logistic regressions within the genotype groups m.3243 A>G, multiple mtDNA deletions, single mtDNA deletion, and primary LHON variants. Multiple imputation by chained equations (R package *mice*) was used resulting in 5 imputed datasets; estimates from imputed datasets were pooled using Rubin rules. OR as well as p values (p) are given separately for imputed and non-imputed (complete cases). In the single mtDNA deletion group, dyslipidemia was significantly associated with mDM in the multiply imputed dataset (OR: 3.81; 95% CI, 1.12-12.99; p=0.033) but not in complete-case analysis (p=0.18). Abbreviations: BMI: body mass index, CI: confidence interval, HR: hazard ratio, OR: odds ratio.

**eFigure 4. Polyneuro-, retino-, and nephropathy in m.3243A>G patients with mDM compared to those without mDM**

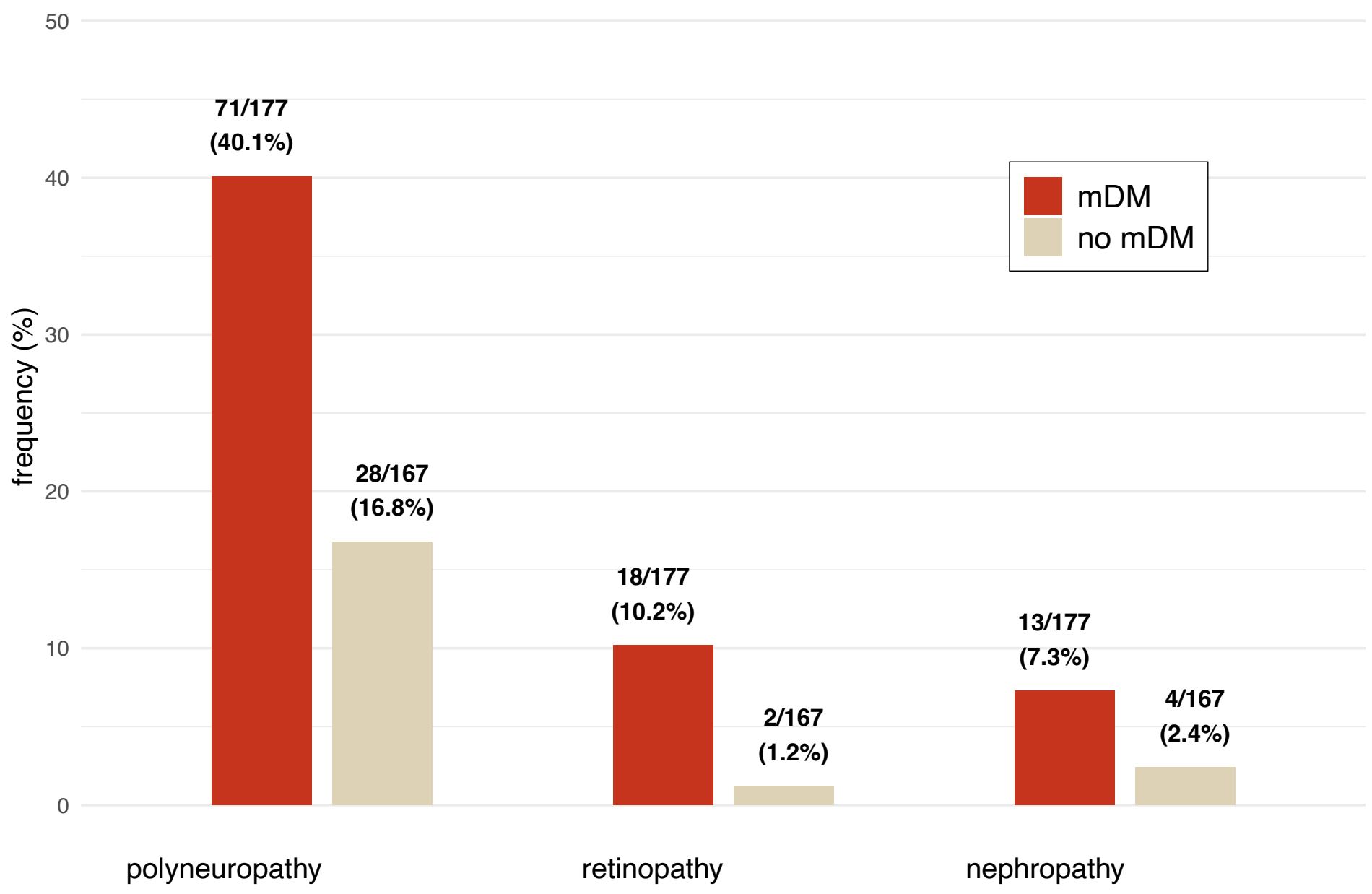


Bar plot indicating the number of mDM patients (red) and non-mDM patients (sand) with the m.3243A>G variant who have polyneuropathy, retinopathy, or nephropathy. The total amount of occurrences within each group is given above the bar along with the respective percentage. Abbreviations: mDM: mitochondrial diabetes mellitus

**eTable 6. Logistic regression on the relevance of mDM status for having complications such as polyneuro-, retino-, and nephropathy**

| **Logistic regression on the occurrence of complications within the m.3243A>G genotype group.** | **Odds Ratio (95%CI)** | **p value** |
|---|---|---|
| mDM presence | 3.72 (1.64 to 8.44) | 0.0019 |

The logistic regression was performed on the imputed m.3243A>G dataset, as indicated in the flowchart eFigure 1. Complication (Polyneuropathy, Nephropathy, and Retinopathy) was used as the dependent variable of interest, whereas the presence of mDM was used as the independent variable; the model was controlled for the covariates sex, age, BMI, dyslipidemia, hypertension, and the age-corrected heteroplasmy level in blood. mDM presence was significantly associated with the occurrence of complications (OR: 3.72 (1.64 to 8.44), p=0.0019) Abbreviations: BMI: body mass index, CI: confidence interval, mDM: mitochondrial diabetes mellitus, OR: odds ratio.

**eFigure 5. Occurrence of mDM and complication diagnosis**

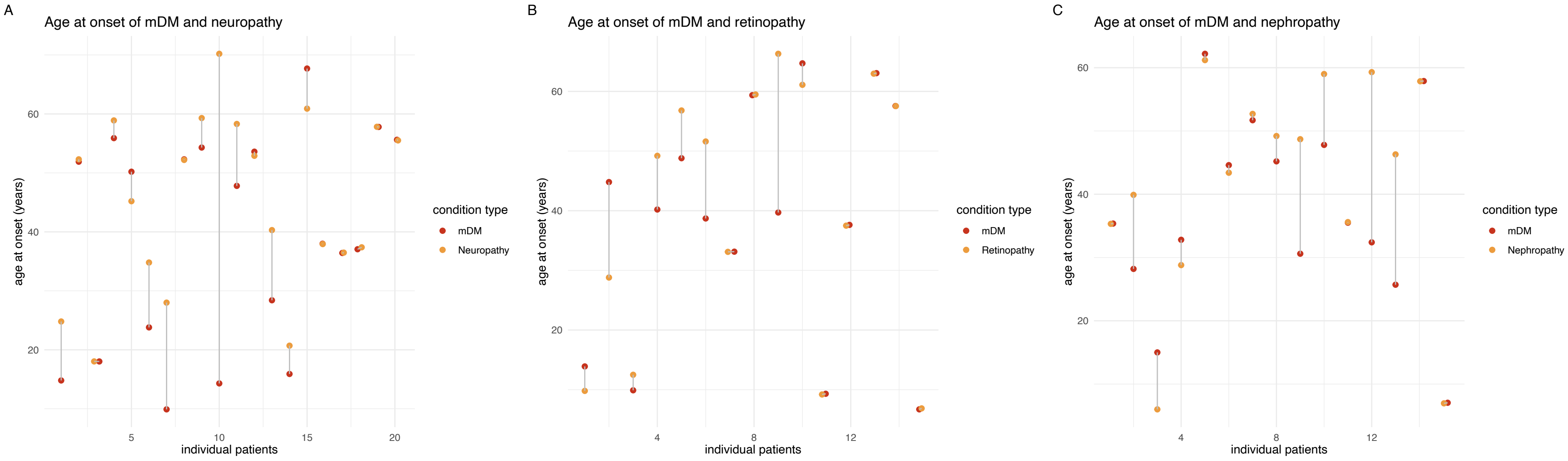


Presentation of the time between mDM onset and the respective complication A polyneuropathy, B retinopathy, or C nephropathy. The orange dots represent onset of polyneuropathy in A, retinopathy in B, and nephropathy in C, while the red dots represent mDM onset in all charts. The line between the points indicate the time difference in years; calculations were performed on an individual level and results are displayed for all patients with both time points available, irrespective of the underlying genotype. Abbreviations: mDM: mitochondrial diabetes mellitus.

**eFigure 6. Prevalence of cardiovascular risk factors dyslipidemia and hypertension in mDM and non-mDM patients**

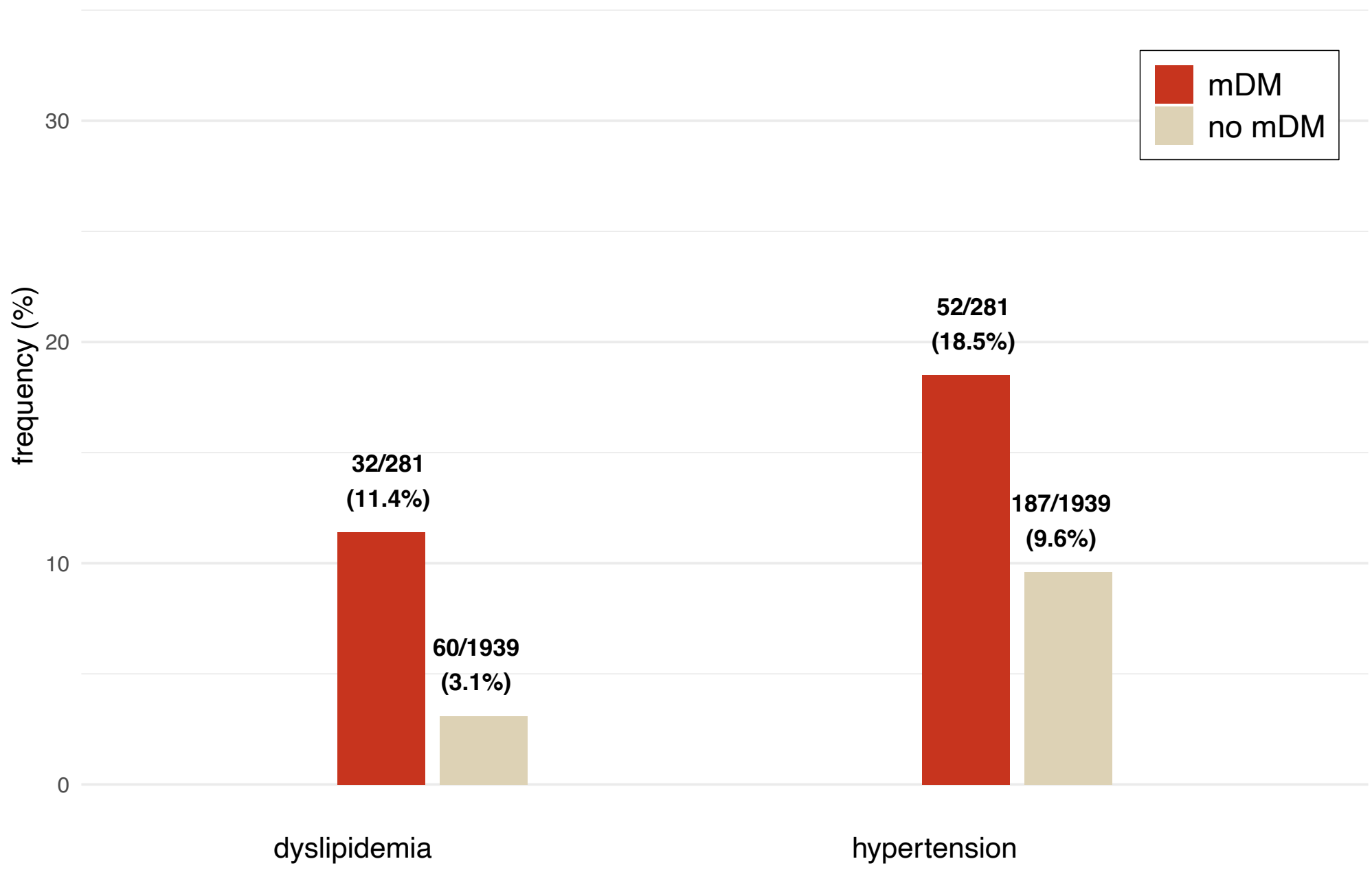


Bar plot indicating the number of mDM patients (red) and non-mDM patients (sand) with the m.3243A>G variant who have dyslipidemia, or hypertension. The total amount of occurrences within each group is given above the bar along with the respective percentage. Abbreviations: mDM: mitochondrial diabetes mellitus.

**eTable 7. Logistic regression analysis on the influence of mDM presence on the likelihood of having hypertension in m.3243A>G patients**

| Logistic regression on the occurrence of hypertension within the m.3243A>G genotype group. | Odds Ratio (95%CI) | p value |
|---|---|---|
| mDM presence | 5.42 (1.08 to 27.21) | 0.040 |

The logistic regression was performed on the imputed m.3243A>G dataset, as indicated in the flowchart eFigure 1. Hypertension was used as the dependent variable of interest, whereas the presence of mDM was used as the independent variable; the model was controlled for the covariates sex, age, BMI, dyslipidemia, and the age-corrected heteroplasmy level in blood. mDM presence was significantly associated with the occurrence of complications (OR: 5.42 (1.08 to 27.21), p=0.040) Abbreviations: BMI: body mass index, CI: confidence interval, mDM: mitochondrial diabetes mellitus, OR: odds ratio.

**eTable 8. Logistic regression analysis on the influence of mDM presence on the likelihood of having dyslipidemia in m.3243A>G patients**

| **Logistic regression on the occurrence of dyslipidemia within the m.3243A>G genotype group.** | **Odds Ratio (95%CI)** | **p value** |
|---|---|---|
| mDM presence | 1.65 (0.47 to 6.56) | 0.47 |

The logistic regression was performed on the imputed m.3243A>G dataset, as indicated in the flowchart eFigure 1. Dyslipidemia was used as the dependent variable of interest, whereas the presence of mDM was used as the independent variable; the model was controlled for the covariates sex, age, BMI, hypertension, and the age-corrected heteroplasmy level in blood. The presence of mDM was not significantly associated with the occurrence of dyslipidemia. Abbreviations: BMI: body mass index, CI: confidence interval, mDM: mitochondrial diabetes mellitus, OR: odds ratio.

**eFigure 7. Longitudinal HbA1c Trajectories Relative to Baseline Measurement to display long-term glycemic control**

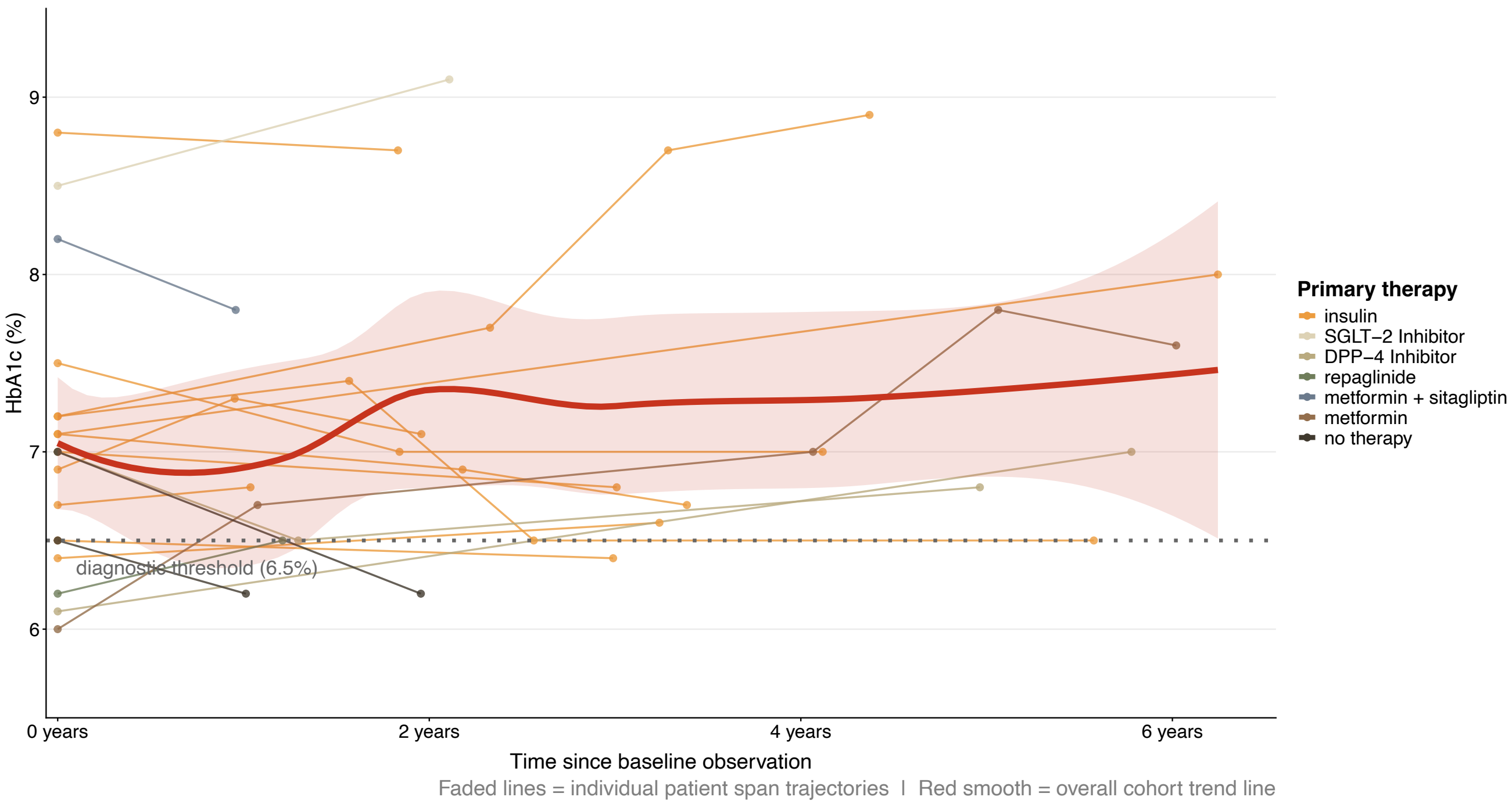


HbA1c measurements over time for individual patients for whom at least two timepoints of measurement were available. The y-axis is showing HbA1c in percent, and the x-axis showing date (individually anchored to initial evaluation as year 0). Points represent HbA1c measurements, colored by primary therapy type. Data include n = 19 patients with n = 45 total HbA1c measurements. Longitudinal data span up to 6.2 years. A LOESS smoothing curve is overlaid to visualize the overall trend in HbA1c over time ranging from 6.9% to 7.5%.

**eFigure 8. PRISMA flow chart summarizing the results of the literature search on antidiabetic drug usage in MD patients**

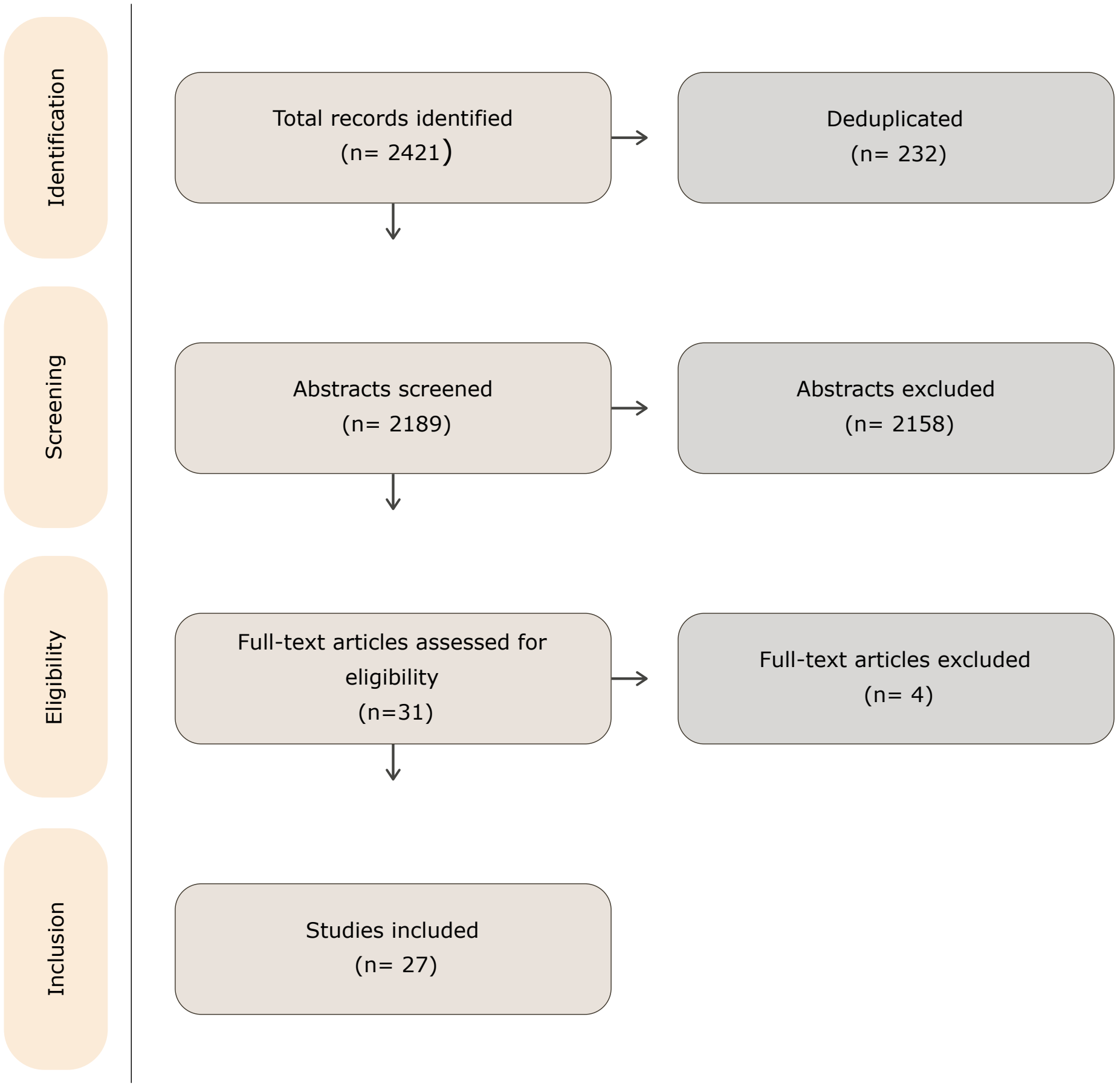


PRISMA flow chart indicating the number of identified total records, deduplicated and excluded abstracts as well as full-text articles. As a result, the total number (n = 27) of included studies is given.

**eTable 9. Results of the literature review on antidiabetic drug usage in MD patients**

| Publication (Author, Year) | Study population | Treatment | Key points of clinical presentation/outcome |
|---|---|---|---|
| Shin 2024[2] | • 32-year-old Korean patient with m.3243A>G and MELAS | • Sulfonylurea<br>• Metformin<br>• DPP4-inhibitor | • poor glycemic control under gliclazide → switch to metformin<br>• after metformin start → Lactic acidosis, progression of focal seizures into refractory status epilepticus, occurrence of dysphagia and progressive multifocal lesions on MRI<br>• switch from metformin to gemigliptin → symptoms subsided |
| Seo 2024[3] | • Patient 1: 48-year-old woman with m.3243A>G and MIDD<br>• Patient 2: 21-year-old man with m.3243A>G and MIDD | • Insulin<br>• DPP-4-inhibitor<br>• Sulfonylurea<br>• Metformin | • patient 1: poor glycemic control with insulin and oral hypoglycemic agents due to incompliance<br>• patient 2: initially good glycemic control on DPP4-inhibitor, sulfonylurea and metformin, 6 years later insufficient glycemic control → additional insulin usage necessary |
| Tran 2023[4] | • 36-year-old woman with m.3243A>G and MELAS | • Insulin<br>• DPP-4-inhibitor | • good glycemic control on insulin and DPP-4 inhibitor<br>• ongoing SLEs, increased seizure activity and rapid decline in consciousness, hearing capacity, and visual acuity after mDM diagnosis |
| Murakami 2023[5] | • 40-year-old man with m.3243A>G and MELAS | • DPP-4-inhibitor<br>• Metformin<br>• SGLT2-inhibitor | • 4 months after metformin start presumably first stroke-like lesion<br>• switch from metformin to DPP4-inhibitor and SGLT2-inhibitor |
| Simoes de Carvalho 2023[6] | • Patient 1: 50-year-old male m.3243A>G and MIDD<br>• Patient 2: 56-year-old male with m.9325T>C and MIDD | • Insulin<br>• DPP-4-inhibitor | • patient 1: 17 years insulin and DPP-4-inhibitor therapy → insufficient glycemic control → additional ultra-rapid insulin → good glycemic control<br>• patient 2: 16 years basal-bolus regimens of insulin → insufficient glycemic control → switch to fast-acting insulin and adaptation of bolus insulin → glucose profile improved |
| Tong 2022[7] | • 16 patients with m.3243A>G and mDM<br>• One 53-year-old woman with m.3243A>G and mDM | • Metformin<br>• Insulin<br>• DPP-4-inhibitor | • 10/16 patients got metformin (60%)<br>• 7 of those 10 patients developed newly neurologic manifestations<br>• The OR was 3.50 [CI 0.37–33.0; p = 0.3287] and not significant<br>• 53-year-old patient: drowsiness, involuntary twitching and abnormal movement occurred 1 month after starting of metformin → stopped after metformin cessation<br>• 12 years satisfactory glycemic control on insulin and DPP-4-inhibitor |
| Lin 2022[8] | • 43-year-old man with m.3243A>G and MELAS | • DPP-4-inhibitor<br>• Metformin | • 2 months after metformin and vildagliptin usage first stroke-like episode<br>• switch from metformin and vildagliptin to linagliptin |
| Zhao 2022[9] | • 12 patients with various mtDNA variants and mitochondrial diabetes (11-50 years of age) | • Metformin<br>• Insulin<br>• GLP-1-agonist<br>• SGLT-2-inhibitor<br>• DPP4-inhibitor | • First visit (before diagnosis of PMD): Metformin was used in 3 patients, 7 needed insulin, in 1 case combined with a DPP4-inhibitor, and in 1 with sulfonylurea<br>• Follow up (longest period was 16 years): 11 patients used insulin, in 6 cases combined with GLP1-agonist, SGLT2-inhibitors, or DPP4-inhibitors; metformin was not used anymore<br>• No adverse events reported |
| Yeung 2021[10] | • Patient 1: 51-year-old man with m.3243A>G and MELAS<br>• Patient 2: 32-year-old woman with m.3243A>G and MIDD<br>• Patient 3: 72-year-old woman with m.3243A>G and MELAS | • Metformin<br>• Sulfonylurea<br>• SGLT2-inhibitor<br>• DPP-4-inhibitor<br>• GLP-1-agonist | • Patient 1: after development of SLE transition of metformin to SGLT2-inhibitor, sulfonylurea and DPP-4-inhibitor → suboptimal glycemic control → switch to GLP-1-agonist → HbA1c improved, no side effects<br>• Patient 2: Metformin and sulfonylurea therapy → suboptimal glycemic control → switch to SGLT2-inhibitor→ HbA1c improved, no side effects<br>• Patient 3: treated with metformin for 5 years without lactic acidosis → after development of SLE MD diagnosis and thus transition to sulfonylurea and DPP-4-inhibitor → insufficient glycemic control → switch to SGLT2-inhibitor and increase of DPP-4-Inhibitor dose → HbA1c improved, no side effects |
| Shin 2021[11] | • 48-year-old woman with m.3243A>G and MIDD | • Metformin<br>• Insulin | • 9 years after mDM diagnosis insufficient glycemic control under oral hypoglycemic agents including metformin → transition to insulin |

| | | | |
|---|---|---|---|
| Lebbar 2021[12] | • 50-year-old female with m.3243A>G and MIDD | • Insulin<br>• Metformin<br>• GLP-1-agonist | • Insulin treatment → insufficient glycemic control → Metformin evoked gastrointestinal side effects → GLP-1-agonist in addition to insulin → HbA1c improved, no side effects |
| Kim 2020[13] | • 27-year-old man with m.3243A>G and MELAS | • Metformin<br>• Sulfonylurea | • After 4 months usage of metformin → first SLE and seizure<br>• Discharged with sulfonylurea |
| Chen 2019[14] | • 52-year-old woman with m.3243A>G and MELAS | • DPP-4-inhibitor<br>• Sulfonylurea<br>• Metformin | • 1 month after adding metformin to the pre-existing oral antidiabetic therapy with sitagliptin and glimepiride occurrence of first SLE and generalized tonic-clonic seizure |
| Keidai 2019[15] | • 72-year-old woman with m.3243A>G and mDM | • Insulin | • Insulin treatment for 30 years<br>• Occurrence of diabetic ketoacidosis due to missed insulin injections during gastroenteritis<br>• As diabetic ketoacidosis improved lactic acidosis developed → resolved after few days |
| Fukuda 2019[16] | • 55-year-old with m.3243A>G and MELAS | • Insulin<br>• Sulfonylurea<br>• DPP-4-inhibitor | • 1 year after mDM diagnosis (age 41) diabetic ketoacidosis occurred → treatment with insulin and sulfonylurea<br>• 12 years later another episode of diabetic ketoacidosis combined with SLE → treatment complemented with DPP-4-inhibitor<br>• 3 months later cessation of sulfonylurea → HbA1c worsened → increasing insulin dose → HbA1c improved |
| Gragera-Martinez 2017[17] | • 55-year-old woman with m.3243A>G and MIDD | • Metformin | • Metformin intake for 30 years<br>• no neurological manifestation or acidotic decompensation reported |
| Murakami 2016[18] | • 24-year-old-woman with m.3243A>G and mDM | • Insulin<br>• GLP-1-agonist | • Initial therapy with insulin → desire of patient to reduce daily injections → adding GLP-1-agonist and reducing daily insulin injections → good glycemic control, no adverse events |
| Ninomiya 2016[19] | • 45-year-old woman with m.3243A>G and MIDD | • Miglitol<br>• Pioglitazone | • Initial diagnosis of gestational diabetes at 41 years of age → well controlled with diet<br>• 4 years later progressive worsening of glycemic control → miglitol start → improved glycemic control, but side effect: diarrhea → pioglitazone, good glycemic control |
| Inoue 2016[20] | • 32-year-old Japanese male with m.14709T>C mutation, mDM and myopathy | • Insulin<br>• Pyruvate | • Insulin-dependent mDM<br>• Additional therapy with sodium-pyruvate → improved insulin secretion, lower insulin dosage per day was necessary<br>• No side effects, however, therapy was terminated after 10 months (severe phenotype, could not self-administer therapy)<br>• Insulin secretion decreased, HbA1c level increased after cessation |
| Naing 2014[21] | • 6 patients with m.3243A>G and MIDD (52-77 years of age) | • Sulfonylurea<br>• Insulin<br>• Metformin | • Index case: 77-year-old woman required insulin within 5 years of initial diagnosis of mDM<br>• 5/6 patients were insulin-dependent<br>• All patients have been treated with metformin at some stage of their disease, side effects were not reported |
| Donovan 2006[22] | • 44-yr-old woman with m.3243A>G and MIDD | • Sulfonylurea<br>• Insulin | • Sulfonylurea poorly tolerated (no details given) → switch to insulin → insufficient glycemic control → start of continuous s.c. insulin infusion device → initially marked improvement, but deterioration again 1 year later |
| Laloi-Michelin 2006[23] | • 43-year-old woman with large mtDNA deletion and KSS | • Metformin<br>• Sulfonylurea<br>• Insulin | • mDM since the age of 19 years → therapy with metformin and sulfonylurea → insufficient glycemic control → switch to Insulin |
| Iwase 2001[24] | • 30-year-old female with m.3243A>G and MELAS | • Insulin | • Occurrence of post-treatment retinopathy due to strict glycemic control |
| Nakamura 1999[25] | • 24-year-old male with m.3243A>G and MIDD | • Insulin | • 1 year after mDM onset: non-ketotic hyperosmolar coma → start insulin → incompliant administration → nephrotic syndrome and insufficient glycemic control |
| Suzuki 1997[26] | • 15 subjects with m.3243A>G | • Insulin<br>• Sulfonylurea | • 11/15 subjects were treated with coenzyme Q10<br>• 8 of them additionally received insulin, 2 were on diet therapy and 1 on sulfonylurea |

| | | | |
|---|---|---|---|
| | | • Coenzym Q10 | • No difference of plasma glucose or HbA1c before and after coenzyme Q10 treatment |
| Suzuki 1995[27] | • 3 patients with m.3243A>G and mDM (51-71 years of age) | • Insulin<br>• Sulfonylurea | • 3 patients have developed leg edema after start of insulin therapy (acute and chronic, starting 1 month up to 2 years after insulin start)<br>• In 2 patients, leg edema has resolved spontaneously, in one under additional coenzyme Q10 therapy<br>• One patient developed hepatic dysfunction 20 days after start of sulfonylurea therapy |
| Katagiri 1994 / Oka 1995[28,29] | • Four pedigrees with m.3243A>G and mDM | • Insulin<br>• Sulfonylurea | • 11/18 patients were treated with sulfonylurea → secondary failure with progression into insulin-dependence after 8 years at the latest |

Abbreviations: BMI: body mass index, DPP-4: dipeptidyl peptidase 4, GLP-1: glucagon-like peptide 1, KSS: Kearns-Sayre syndrome, mDM mitochondrial diabetes mellitus, MELAS: mitochondrial encephalopathy, lactic acidosis and stroke-like episodes, MIDD: maternally inherited diabetes and deafness, MD: mitochondrial disease, NMDAS: Newcastle Mitochondrial Disease Adult Scale, OR: odds ratio, SLE: stroke-like episode, SGLT-2: sodium-glucose cotransporter 2.

## References


1 Schaefer, A. M. et al. Mitochondrial Disease in Adults: A Scale to Monitor Progression and Treatment. *Neurology* **66**, 1932–1934 (2006).

2 Shin, H. J., Na, J.-H. & Lee, Y.-M. A Case of Exacerbated Encephalopathy with Stroke-Like Episodes and Lactic Acidosis Triggered by Metformin in a Patient with Melas. *Neurological Sciences* **45**, 2337–2339 (2024).

3 Seo, J.-S. Chronological Change of Left Ventricular Global Longitudinal Strain in Patients with Maternally Inherited Diabetes and Deafness: A Case Series. *Medicine* **103** (2024).

4 Tran, N. Q., Phan, C. C., Vuong, T. B., Tran, T. V. & Ma, P. T. Acute Onset of Diabetes and Rapid Cognitive Decline in a Patient with Mitochondrial Encephalomyopathy, Lactic Acidosis, and Stroke-Like Episodes Syndrome. *Endocrinology, Diabetes & Metabolism Case Reports* **2023**, 22–0416 (2023).

5 Murakami, K., Sakamoto, K., Ishiguchi, H. & Ito, H. Mitochondrial Encephalopathy, Lactic Acidosis, and Stroke-Like Episodes Diagnosed after Metformin-Triggered Stroke-Like Episodes. *J Stroke Cerebrovasc Dis* **32**, 107080 (2023).

6 Simões de Carvalho, F., de Brito Marques, F., Lopes, A. E., Lima Ferreira, J. & Carneiro de Melo, P. Ultra-Rapid Insulin in Mitochondrial Diabetes: Two Clinical Cases. *Endocrinología, Diabetes y Nutrición (English ed)* **70**, 364–366 (2023).

7 Tong, H.-F. et al. Neurological Manifestations in M.3243a>G-Related Disease Triggered by Metformin. *Journal of Diabetes and its Complications* **36**, 108111 (2022).

8 Lin, W.-H., Yang, I.-H., Cheng, H.-E. & Lin, H.-F. Case Report: Late-Onset Mitochondrial Disease Uncovered by Metformin Use in a Patient with Acute Verbal Auditory Agnosia. *Frontiers in Neurology* **13** (2022).

9 Zhao, Y. et al. The Role of Lactate Exercise Test and Fasting Plasma C-Peptide Levels in the Diagnosis of Mitochondrial Diabetes: Analysis of Clinical Characteristics of 12 Patients with Mitochondrial Diabetes in a Single Center with Long-Term Follow-Up. *Frontiers in Endocrinology* **13** (2022).

10 Yeung, R. O. et al. Management of Mitochondrial Diabetes in the Era of Novel Therapies. *J Diabetes Complications* **35**, 107584 (2021).

11 Shin, Y. H. et al. Concomitant Mitochondrial Diabetes and Myopathy Mistook for Complications of Immunosuppressants after Kidney Transplant. *Exp Clin Transplant* **19**, 736–738 (2021).

12 Lebbar, M., Timsit, J., Luyton, C. & Marchand, L. Glucagon-Like Peptide-1 Receptor Agonists (Glp1-Ra) in the Treatment of Mitochondrial Diabetes. *Acta Diabetologica* **58**, 1281–1282 (2021).

13 Kim, N. H., Siddiqui, M. & Vogel, J. Melas Syndrome and Midd Unmasked by Metformin Use: A Case Report. *Annals of Internal Medicine* **174**, 124–125 (2020).

14 Chen, W. T., Lin, Y. S., Wang, Y. F. & Fuh, J. L. Adult Onset Melas Syndrome Presenting as a Mimic of Herpes Simplex Encephalitis. *Acta Neurol Taiwan* **28(2)**, 46–51 (2019).

15 Keidai, Y. et al. “Switched” Metabolic Acidosis in Mitochondrial Diabetes Mellitus. *Journal of Diabetes Investigation* **10**, 1116–1117 (2019).

16 Fukuda, M. & Nagao, Y. Dynamic Derangement in Amino Acid Profile during and after a Stroke-Like Episode in Adult-Onset Mitochondrial Disease: A Case Report. *Journal of Medical Case Reports* **13**, 313 (2019).

17 Gragera-Martínez, Á., Fernández-González, G. & León-Justel, A. Maternally Inherited Diabetes and Deafness in 4 Family Members with DNA Mutation and at Least 4 Generations with Suggestive Disease Phenotype. *The Journal of Applied Laboratory Medicine* **2**, 278–283 (2017).

18 Murakami, T. et al. Successful Glycemic Control Decreases the Elevated Serum Fgf21 Level without Affecting Normal Serum Gdf15 Levels in a Patient with Mitochondrial Diabetes. *The Tohoku Journal of Experimental Medicine* **239**, 89–94 (2016).

19 Ninomiya, H. et al. Treatment of Mitochondrial Diabetes with a Peroxisome Proliferator-Activated Receptor (Ppar)-Gamma Agonist. *Internal Medicine* **55**, 1143–1147 (2016).

20 Inoue, T. et al. Pyruvate Improved Insulin Secretion Status in a Mitochondrial Diabetes Mellitus Patient. *J Clin Endocrinol Metab* **101**, 1924–1926 (2016).

21 Naing, A. et al. Maternally Inherited Diabetes and Deafness (Midd): Diagnosis and Management. *Journal of Diabetes and its Complications* **28**, 542–546 (2014).

22 Donovan, L. E. & Severin, N. E. Maternally Inherited Diabetes and Deafness in a North American Kindred: Tips for Making the Diagnosis and Review of Unique Management Issues. *The Journal of Clinical Endocrinology & Metabolism* **91**, 4737–4742 (2006).

23 Laloi-Michelin, M. et al. Kearns Sayre Syndrome: An Unusual Form of Mitochondrial Diabetes. *Diabetes & Metabolism* **32**, 182–186 (2006).

24 Iwase, M., Gotoh, D., Ishibashi, T., Yoshinari, M. & Fujishima, M. Post-Treatment Retinopathy in a Case of Diabetes Mellitus with A3243g Mutation of Mitochondrial DNA. *J Diabetes Complications* **15**, 167–169 (2001).

25 Nakamura, S. et al. Renal Complications in Patients with Diabetes Mellitus Associated with an a to G Mutation of Mitochondrial DNA at the 3243 Position of Leucine Trna. *Diabetes Research and Clinical Practice* **44**, 183–189 (1999).

26 Suzuki, Y. et al. Diabetes Mellitus Associated with 3243 Mitochondrial Trna(Leu(Uur)) Mutation: Clinical Features and Coenzyme Q10 Treatment. *Mol Aspects Med* **18 Suppl**, S181–188 (1997).

27 Suzuki, Y. et al. Insulin Edema in Diabetes Mellitus Associated with the 3243 Mitochondrial Trna<Sup>Leu(Uur)</Sup> Mutation; Case Reports. *Diabetes Research and Clinical Practice* **29**, 137–142 (1995).

28 Katagiri, H. et al. Mitochondrial Diabetes Mellitus: Prevalence and Clinical Characterization of Diabetes Due to Mitochondrial Trnaleu(Uur) Gene Mutation in Japanese Patients. *Diabetologia* **37**, 504–510 (1994).

29 Oka, Y. et al. Mitochondrial Diabetes Mellitus—Glucose-Induced Signaling Defects and β-Cell Loss. *Muscle & Nerve* **18**, S131–S136 (1995).

## B GENOMIT Study group

| First Names | Surnames |
|---|---|
| Enrico | Bertini |
| Almut T. | Bischoff |
| Nikolas | Boy |
| Claudio | Bruno |
| Valerio | Carelli |
| Giulia | Cecchi |
| Kristl | Claeys |
| Felix | Distelmaier, |
| Massimiliano | Filosto |
| Caterina | Garone |
| Maja | Hempel |
| Daniela | Karall |
| Chirstian | Kmop |
| Rania | Kotzaeridou |
| Piervito | Lopriore |
| Tiziana | Mongini |
| Vincenzo | Montano |
| Olimpia | Musumeci |
| Valeria | Nicoletta |
| Guido | Primiano |
| Elena | Procopio |
| Rita | Rinaldi |
| Lucia | Ruggiero |
| René | Santer |
| Heinrich | Sasse |
| Jochen | Schäfer |
| Christian | Schlein |
| Ludger | Schöls |
| David | Strube |
| Annemarie | Thaele |
| Maria Lucia | Valentino |
| Jürgen-Christoph | von Kleist |
| Leopold | Zeng |
